# Restoring adapter protein complex 4 function with small molecules: an in silico approach to spastic paraplegia 50

**DOI:** 10.1002/pro.70006

**Published:** 2024-12-26

**Authors:** Serena Francisco, Lorenzo Lamacchia, Attilio Turco, Giuseppe Ermondi, Giulia Caron, Matteo Rossi Sebastiano

**Affiliations:** ^1^ Department of Molecular Biotechnology and Health Sciences University of Torino Torino Italy

**Keywords:** AP‐4, AP4M1, drug repurposing, druggability, hereditary spastic paraplegia, ligandability, molecular modeling, personalized medicine, rare diseases, SPG50

## Abstract

This study focuses on spastic paraplegia type 50 (SPG50), an adapter protein complex 4 deficiency syndrome caused by mutations in the adapter protein complex 4 subunit mu‐1 (*AP4M1*) gene, and on the downstream alterations of the AP4M1 protein. We applied a battery of heterogeneous computational resources, encompassing two in‐house tools described here for the first time, to (a) assess the druggability potential of AP4M1, (b) characterize SPG50‐associated mutations and their 3D scenario, (c) identify mutation‐tailored drug candidates for SPG50, and (d) elucidate their mechanisms of action by means of structural considerations on homology models of the adapter protein complex 4 core. Altogether, the collected results indicate R367Q as the mutation with the most promising potential of being corrected by small‐molecule drugs, and the flavonoid rutin as best candidate for this purpose. Rutin shows promise in rescuing the interaction between the AP4M1 and adapter protein complex subunit beta‐1 (AP4B1) subunits by means of a glue‐like mode of action. Overall, this approach offers a framework that could be systematically applied to the investigation of mutation‐wise molecular mechanisms in different hereditary spastic paraplegias, too.

## 1 INTRODUCTION

Rare diseases (RDs) include about 7000 heterogeneous conditions collectively affecting 10% of the global population (Amberger et al., 2015). Unfortunately, there is no treatment available for 95% of these disorders (Roessler et al., 2021). This is due to various reasons: developing a new treatment is expensive, the destination market is small, and the scientific knowledge about rare conditions is limited (Sun et al., 2017). It follows that pharmaceutical companies tend to overlook RDs to focus on more prevalent illnesses. Because of this, more feasible drug discovery campaigns are urgently needed to support RD patients and their families.

Hereditary spastic paraplegias (HSPs) are rare neurodegenerative disorders with either autosomal dominant, autosomal recessive, X‐linked recessive, or mitochondrial inheritance (Meyyazhagan & Orlacchio, 2022). HSPs are monogenic disorders, and more than 80 gene loci have been linked to HSP phenotypes. The main features of HSPs are gradual lower limb spasticity and weakness, although complex forms of HSP include symptoms such as visual impairment, epilepsy and intellectual disability (Meyyazhagan & Orlacchio, 2022).

Adapter protein complex 4 (AP‐4)‐related HSPs are ultrarare HSPs caused by bi‐allelic loss‐of‐function mutations to genes encoding for the subunits of the AP‐4 complex (Ebrahimi‐Fakhari et al., 2020). These genes include adapter protein complex subunit beta‐1 (*AP4B1*), adapter protein complex 4 subunit mu‐1 (*AP4M1*), adapter protein complex subunit epsilon (*AP4E1*), and adapter protein complex subunit sigma‐1 (*AP4S1*), which encode for the AP4B1, AP4M1, AP4E1, and AP4S1 protein subunits, respectively (Mattera et al., 2017). It follows that four types of AP‐4‐associated spastic paraplegia do exist, all of them with autosomal recessive inheritance pattern: spastic paraplegia 47 (SPG47)/*AP4B1*, spastic paraplegia 50 (SPG50)/*AP4M1*, spastic paraplegia 51 (SPG51)/*AP4E1*, and spastic paraplegia 52 (SPG52)/*AP4S1*.

In this study, we focus on the SPG50/*AP4M1* pair. SPG50 is characterized by early infantile hypotonia that gradually progresses to spastic tetraplegia, along with developmental delay, intellectual disability, and microcephaly (Brent & Deng, 2023; Ebrahimi‐Fakhari et al., 1993). About 60 SPG50 patients worldwide have been diagnosed so far (https://clinicaltrials.gov/study/NCT05518188. Accessed November 29, 2023), although this number is expected to be higher. The only pharmacological treatment available today for SPG50 is MELPIDA, an SPG50‐targeted gene therapy now in a clinical trial (NCT06069687) (Chen et al., 2023; Dowling et al., 2024). Parallelly, a recent paper reported a lead compound isolated from phenotypic screening able to restore AP‐4‐related functions in fibroblasts and pluripotent stem cell‐derived neurons from AP‐4‐HSP patients (Saffari et al., 2024).

**FIGURE 1:**
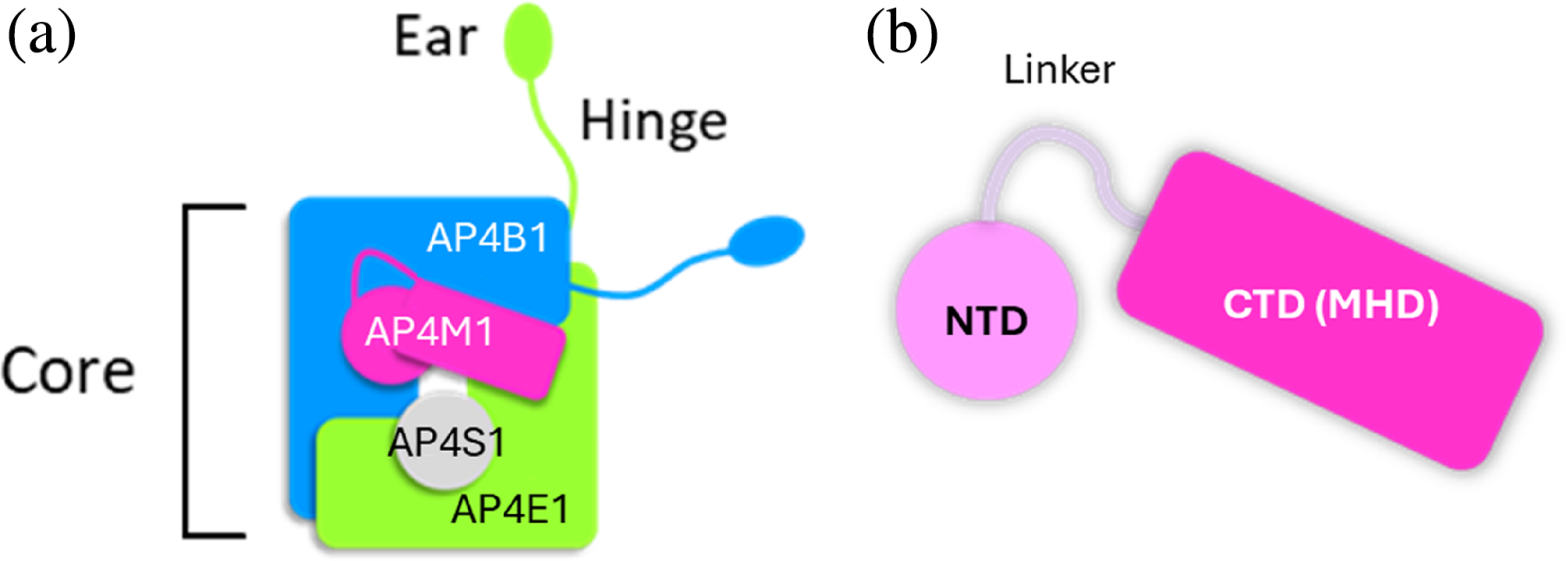
The adapter protein complex 4 (AP‐4) complex and adapter protein complex 4 subunit mu‐1 (AP4M1). (a) Schematic illustration of the adapter protein complex 4. It is formed by two large subunits (AP4B1, in blue, and AP4E1, in green), a medium subunit (AP4M1, in pink), and a small subunit (AP4S1, gray). (b) Structural organization of the AP4M1 subunit: This protein consists of an N‐terminal region (NTD, residues 1–118), a linker, and a C‐terminal domain (CTD), also called mu homology domain (MHD, residues 184–453). AP4B1, adapter protein complex subunit beta‐1; AP4E1, adapter protein complex subunit epsilon; AP4S1, adapter protein complex subunit sigma‐1.

The SPG50 causative gene, *AP4M1*, encodes for the AP‐4 subunit mu‐1 (AP4M1), the medium subunit of the AP‐4 assembly. AP4M1 participates in the formation of the AP‐4 complex together with two large subunits (AP‐4 subunits beta‐1 and epsilon‐1, AP4B1 and AP4E1, respectively) and a small subunit (AP‐4 subunit sigma‐1, AP4S1). The AP‐4 complex is a heterotetrameric protein assembly mediating vesicular trafficking of transmembrane proteins from the *trans*‐Golgi network toward peripheral membrane compartments within eukaryotic cells (Mattera et al., 2020). AP‐4 is organized into different structural domains (Figure 1a): a core domain (responsible for cargo recognition and membrane recruitment), a hinge domain, and an ear domain (involved in the interaction with accessory and/or regulatory proteins) (Park & Guo, 2014). Based on its similarity to other adapter protein complexes (namely, AP‐1 and AP‐2), AP‐4 is expected to adopt two alternative conformations: a closed/inactive state and an open/active one (Canagarajah et al., 2013). The latter is able to interact with target membranes and cargo proteins. Given the homology with the other AP complexes, the tetramerization process is supposed to follow a stepwise assembly, where two hemicomplexes are formed separately (AP4B1‐AP4M1 and AP4E1‐AP4S1) and later interact with each other to form the full multimer (Gulbranson et al., 2019; Mattera et al., 2022; Wan et al., 2021).

AP4M1 is a 453‐amino acid protein consisting of an N‐terminal domain (1–118 ca.) and a C‐terminal mu homology domain (MHD, 184–453) joined by a flexible linker (Figure 1b). The AP4M1‐MHD plays a pivotal role in mediating the recognition of tyrosine‐based sorting signals on cargo proteins when the complex adopts its open conformation (Bonifacino & Dell'Angelica, 1999; Burgos et al., 2010). On the other hand, the amino‐terminal domain (NTD) is expected to dock in the angular bend of the AP4B1 subunit to form one hemicomplex, while the AP4S1 subunit (which displays remarkable structural similarity with the AP4M1‐NTD) docks into the AP4E1 subunit to give rise to the second hemicomplex (Mattera et al., 2022). Although an experimental structure of the AP‐4 complex has not yet been determined, an attempt at in silico structural characterization of AP‐4 and associated HSP‐causing missense variants has previously been carried out by Gadbery and colleagues (Gadbery et al., 2020). In this study, the authors mapped known AP‐4 variants onto a homology model of the complex and evaluated the conservation across evolution of different AP‐4 regions. It highlighted the critical role of residues located at inter‐subunit interfaces in stabilizing the AP‐4 core and pointed out that both the NTD and carboxy‐terminal domain (CTD) of AP4M1 host pathogenic variants. Their work, together with prior literature including publications from our group (Rossi Sebastiano et al., 2022; Rossi Sebastiano et al., 2022; Rossi Sebastiano et al., 2024), underlines the importance of computational approaches relying upon AP‐4 a 3D models to investigate variant‐related effects in the context of AP‐4‐HSPs.

Despite the availability of some structural information on AP‐4 and AP4M1 from homology and crystallographic experiments, no structure‐based, small‐molecule drug repurposing for SPG50 has been attempted so far. Drug repurposing (i.e., rewiring a therapeutic agent toward a different indication than the original one) is a promising strategy (Hechtelt Jonker et al., 2023; Roessler et al., 2021; Shah et al., 2021). It displays compelling advantages (namely, time and cost) and exhibits the potential to fill the therapeutic gap in RD management wherever genetic treatments are not available yet. Moreover, since SPG50 is caused either by homozygous or compound heterozygous mutations in the *AP4M1* gene, correcting at least one out of two variants is expected to suffice to rescue the altered phenotype (Brent & Deng, 2023).

Nevertheless, drug repurposing cannot be blindly applied to every rare condition. It is crucial to thoroughly evaluate the feasibility of drug repurposing campaigns by addressing each case separately in its own specificity and complexity. This evaluation step can be enhanced by the effective combination of computational tools, as highlighted in a previous report from our group (Rossi Sebastiano et al., 2024) and demonstrated in a personalized drug repurposing pipeline for infantile ascending HSP (Rossi Sebastiano et al., 2022).

In this study, we aimed at (a) assessing the druggability potential of AP4M1, (b) characterizing SPG50‐associated mutations and their 3D surrounding, (c) discovering small‐molecule drug candidates for SPG50, and (d) elucidating their mechanism of action through structural considerations based on the AP‐4 core as well. To achieve these goals, we employed a combination of web servers and standalone software that, to our knowledge, have never been integrated before. We first implemented a new platform (named Drug Repurposing Assessment for RD Targets [DRARDT]) to assess the feasibility of a drug repurposing program for SPG50. Then, we mapped SPG50‐associated missense mutations to the structure of AP4M1 by using an in‐house web server presented here for the first time (3DVarPro). Afterward, we performed a structure‐based characterization of a subset of SPG50 variants within the AP‐4 core to ultimately isolate those eligible for targeted drug repurposing. Finally, we hypothesized a molecular mechanism explaining the pathogenic effect downstream of the targeted variant, R367Q, and a mechanism of rescue mediated by a potential drug candidate isolated from virtual screening.

Overall, this study showcases that molecular modeling resources can foster research on RD targets and drive small‐molecule drug repurposing initiatives toward patient‐tailored therapies. Although this work focused on a single case of SPG50, the relevance of our pipeline should be considered in a broader context, where other RDs and mutations could also benefit from the same approach.

## 2 RESULTS

### 2.1 SPG50‐associated missense mutations on the AP4M1 3D structure

**FIGURE 2:**
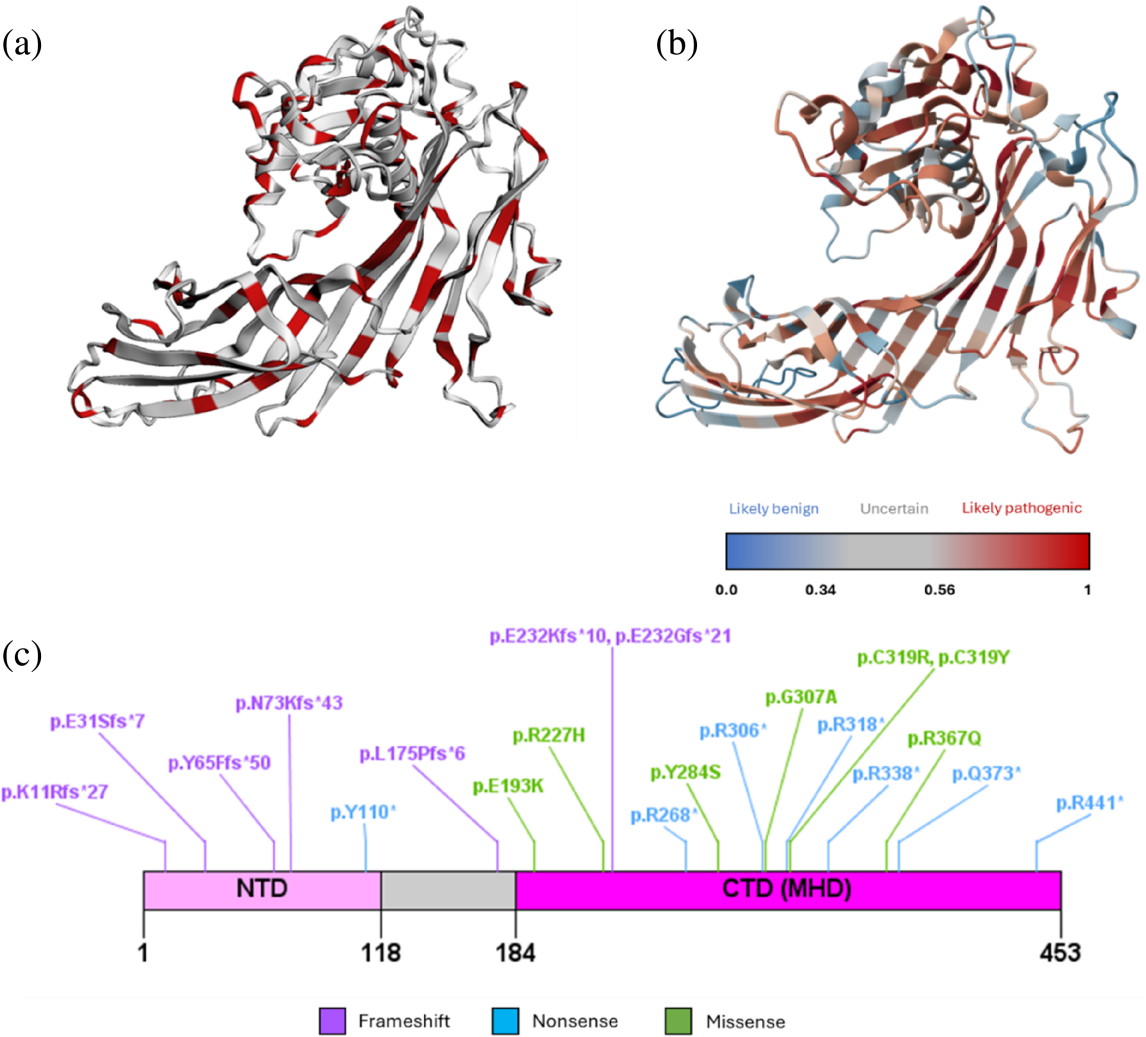
(a) Location of hereditary spastic paraplegia‐associated ClinVar missense variants (in red) on the AlphaFold structure of adapter protein complex 4 subunit mu‐1 (AP4M1). (b) AlphaFold structure of AP4M1 colored by AlphaMissense pathogenicity score. The displayed color for each residue is the average pathogenicity score across all possible amino acid substitutions at the given position. (c) Distribution of AP4M1 protein variants from the dataset of Ebrahimi‐Fakhari and colleagues all over the primary structure of AP4M1.

To gain insight into the spatial distribution of all reported SPG50‐associated missense mutations over the AP4M1 structure, we used our in‐house web app 3DVarPro. 3DVarPro was developed through the Streamlit framework to map all clinical trait‐associated missense mutations retrieved from the ClinVar database onto the wild‐type, 3D structure of a protein of interest automatically retrieved from the Google DeepMind's AlphaFold database (Varadi et al., 2022). This quick visualization highlighted that SPG50 missense mutations are evenly spread across the full‐length structure of AP4M1 (Figure 2a). This finding suggests that the whole subunit is susceptible to functional impairment depending on the mutation. Indeed, previous evidence suggests that mutations of the NTD (Figure 1b) could affect the correct docking of this domain in the AP4B1 subunit, while variants located in the CTD might impair cargo recognition (Gadbery et al., 2020).

We compared the 3DVarPro map of AP4M1 with the one generated by the newly released AphaMissense prediction model (Figure 2b, see Section 4) (Cheng et al., 2023), which confirmed that highly pathogenic missense variants are homogenously spread throughout the AP4M1 structure.

Altogether, these observations suggest that different pathogenic mechanisms may involve AP4M1 and be responsible for SPG50 insurgence, with each one worth being investigated.

### 2.2 Application of DRARDT to assess the feasibility of a drug repurposing program for SPG50

**FIGURE 3:**
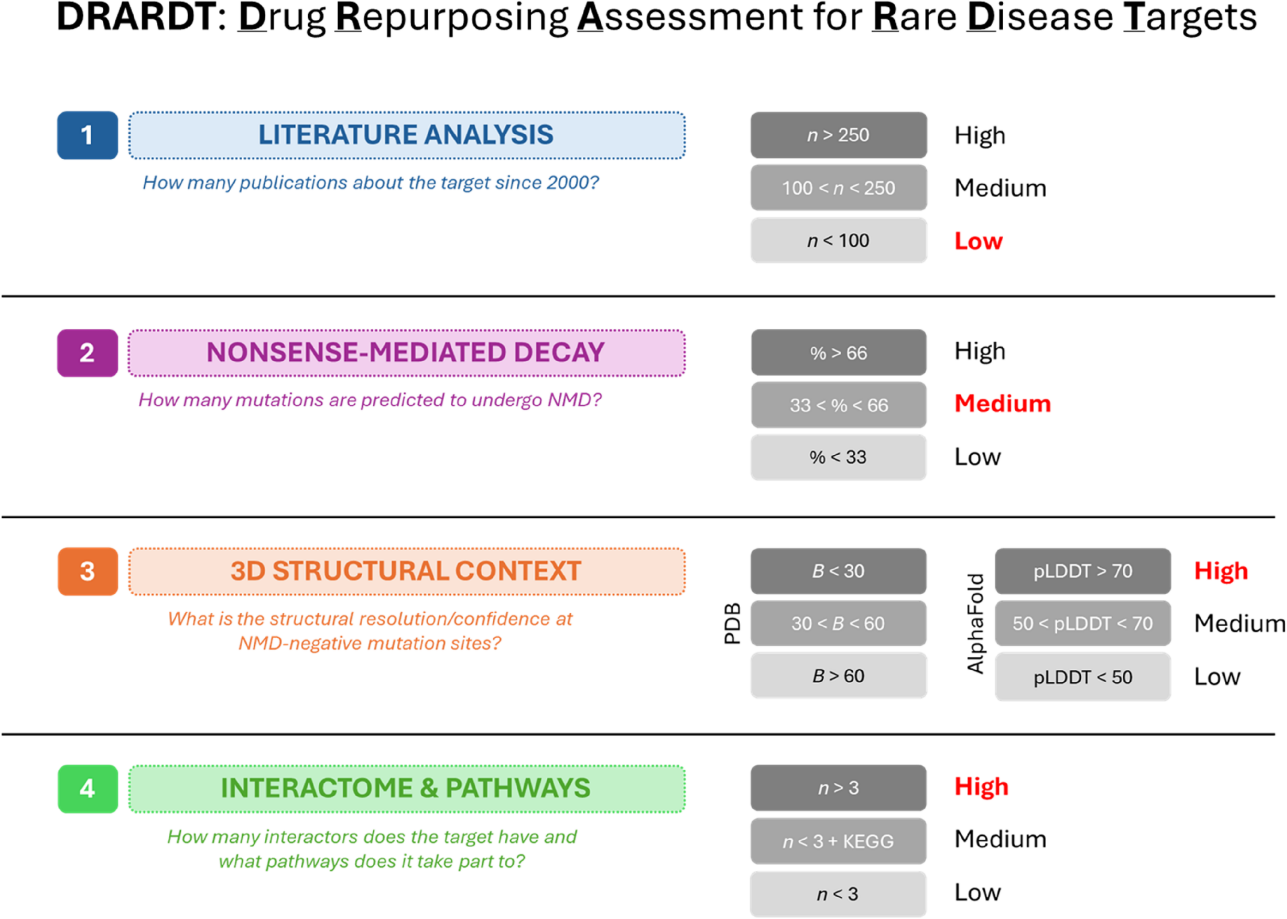
General scheme of Drug Repurposing Assessment for rare diseases (RDs) targets (adapter protein complex 4 subunit mu‐1 outcomes for each criterium are highlighted in bold red characters). (1) Number of publications on PubMed featuring the target. (2) Percentage of RD‐related mutations predicted to undergo NMD. (3) Structural features at NMD‐negative mutant residues. Both available experimental and predicted structures are considered, in which the readout is represented by B‐factor and pLDDT in a 5 Å‐radius area from the mutant site, respectively. (4) Number of interactors and annotated pathways featuring the target.

DRARDT is an innovative methodology developed by our research group. It integrates various types of information to evaluate the feasibility of drug repurposing programs for RD targets. A general scheme of the pipeline is reported in Figure 3, while technical details can be found in Section 4.

DRARDT considers four aspects: (1) the available scientific literature about the target, (2) whether disease‐associated mutations affect protein production, (3) the quality of 3D models at mutation sites, and (4) the possible disruption of protein–protein interaction (PPI) networks upon mutation. Based on the outcome, indexes ranging from “High” (best outcome), to “Medium” and “Low” (worst outcome) are assigned.

Literature analysis consisted of collecting all available publications on the target since 2000. In the case of AP4M1, only 52 publications were highlighted in the 2000–2024 timespan, therefore a “Low” index was assigned to the target. The literature analysis allowed us to identify the pool of SPG50‐associated mutations that were used for the next steps of the investigation. This set was collected from a work by Ebrahimi‐Fakhari et al. (2020) and included 25 univocal mutations (Figure 2c). The considered mutations are seven nonsense, seven frameshift, seven missense, and four variants with unknown effects at the protein level (Table S1). We chose to address published mutations instead of larger archives (e.g., ClinVar) to perform a more clinically relevant analysis, with precise attribution to patient cases and phenotypic implications.

Inference about the presence of mutant protein products was made by predicting the probability of nonsense‐mediated mRNA decay (see Section 4). Out of the seven frameshift variants, four were discarded because their NMD predictions showed low confidence (i.e., no flagged premature termination codon was found to exist in the reading frame). The four unknown (intronic) variants were excluded as well, and 10 out of the remaining 17 mutations (three frameshift, seven nonsense) were predicted to lead to NMD. Only missense mutations passed this selection, accounting for slightly more than 33% of the considered ones (which did not include the four unknown variants). Thus, the NMD‐susceptibility of SPG50‐associated mutations was assigned a “Medium” index (33 < % < 66).

For the third step of DRARDT (i.e., 3D structural information at mutation sites), both experimental (PDB 3L81) and computational (AlphaFold database, UniProt code O00189) 3D structures of AP4M1 were considered. Given that the AlphaFold structure of AP4M1 encompasses the complete amino acid sequence, whereas the crystal structure is limited to the CTD/MHD, both structures were independently assessed for score assignment. This approach acknowledges the non‐overlapping nature of the information provided by each structure, ensuring a comprehensive evaluation. A detailed explanation of how the definition of each mutation site was evaluated is reported in the Section 4 section. In both the experimental and predicted structure of AP4M1, most residues surrounding (and including) the mutation sites were assigned a “High” label. This indicates an overall high structural definition.

Finally, analysis of PPIs and pathways revealed 10 STRING‐DB interactors and 1 KEGG cellular pathway for AP4M1. The considered STRING interactors were either from directly annotated experiments or from linked databases. Therefore, a “High” flag was assigned to AP4M1 for this fourth aspect as well.

In conclusion, despite being a RD target, AP4M1 turned out to be a well‐annotated protein based on our DRARDT evaluation method (Figure 3). As all missense variants are expected to be translated at the protein level, we focused on these mutations for the following stages of this study.

### 2.3 Assessment and prioritization of pathological missense mutations

A key requirement to perform drug repurposing on single residue variants (SRVs) is that they can be targeted by small molecule ligands (ligandability). This lays the foundation for druggability (i.e., that such interaction leads to a desired biological effect) (Di Palma et al., 2023). In practical terms, the difficulty of identifying a molecule to correct a specific SRV can vary depending on the SRV's characteristics, and, in some instances, it may not be possible. In parallel, it is important to formulate hypotheses on the mechanisms downstream of any pathogenic variant to understand whether single mutations can benefit from pharmacological intervention, thus prioritizing SRVs eligible for drug repurposing. To address this question and suggest possible intervention plans, each missense variant was analyzed using a pool of in silico tools enabling the gathering of information about: (1) entity of pathogenicity of SRVs and (2) evolutionary conservation of wild‐type residues, (3) structural order/disorder of AP4M1 regions including residues undergoing mutation, (4) exposure to the solvent, and (5) effect of the various SRVs on the thermodynamics of the protein (i.e., stability and folding) (Table 1).

**TABLE 1:** List of selected tools for the evaluation of “ligandable” SPG50‐associated missense mutations.

Tested aspect	Tools	Description	Link
Pathogenicity	MutPred2	Web app to classify amino acid substitutions as benign or pathogenic	http://mutpred.mutdb.org/
	E‐SNP&GO	ML method to call benign versus pathogenic amino acid substitutions	https://esnpsandgo.biocomp.unibo.it/
Conservation	ConSurf	Web server that infers the evolutionary conservation of either amino or nucleic acid positions in protein/RNA/DNA molecules	https://consurf.tau.ac.il/consurf_index.php
Disorder	IUPred2A	Web interface that generates energy estimation‐based predictions for ordered and disordered protein residues (IUPred2) and for disordered binding regions (ANCHOR2)	https://iupred2a.elte.hu/
Solvent accessibility	FreeSASA	Command line tool to retrieve the solvent‐accessible surface area or residues within a protein (PDB) structure	https://freesasa.github.io/
Stability	DynaMut2	Web interface and command line tool for the assessment of changes in protein stability and flexibility upon missense mutations	https://biosig.lab.uq.edu.au/dynamut2/
	SimBaNI	Multilinear regression model to predict protein stability changes upon missense mutation	–
	INPS‐3D	Web server for the prediction of the impact of missense mutations on protein stability	https://inps.biocomp.unibo.it/inpsSuite/default/index3D

**FIGURE 4:**
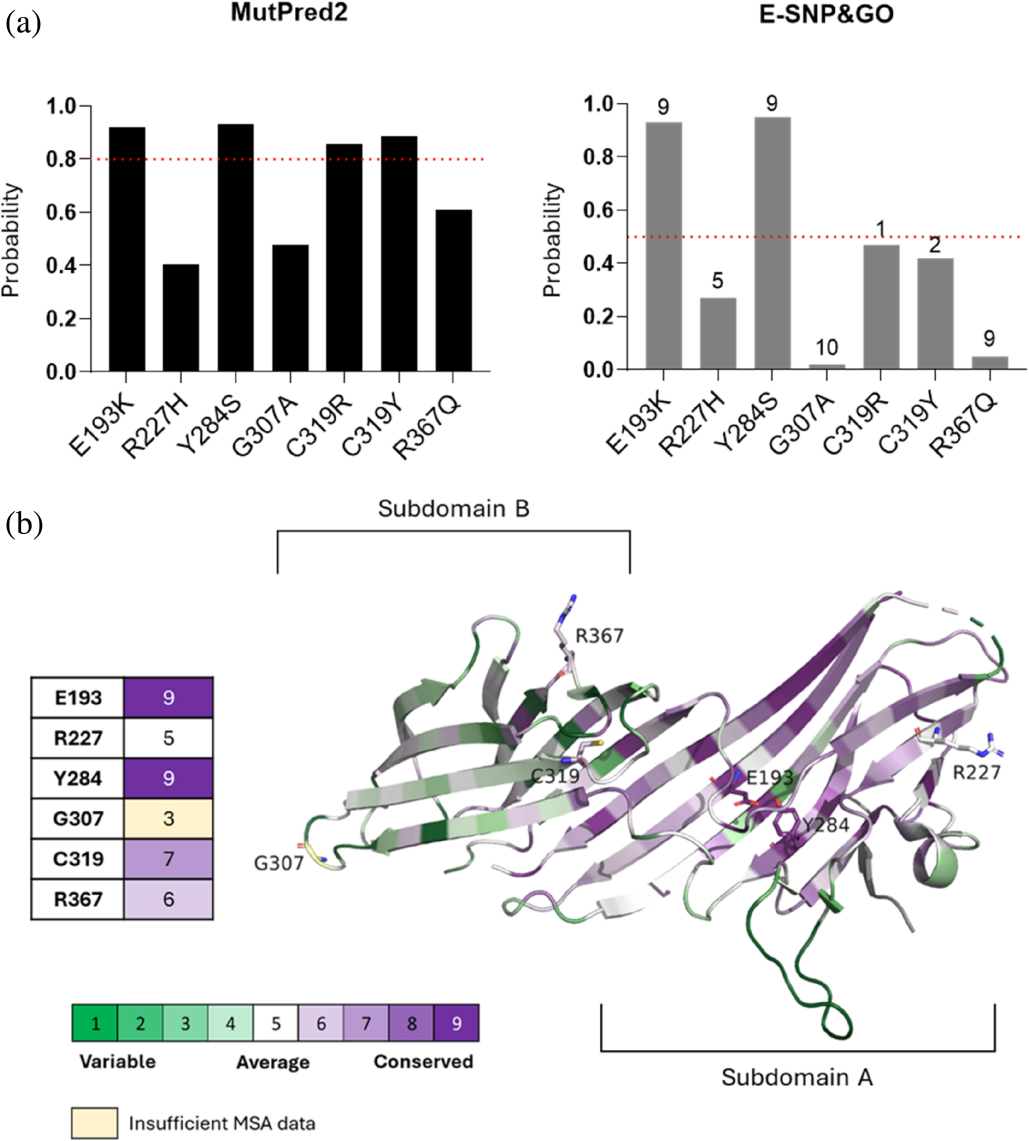
Pathogenicity and evolutionary conservation assessment of spastic paraplegia type 50‐associated missense mutations. (a) Probability of pathogenicity for each missense variants predicted by the MutPred2 and E‐SNP&GO web servers. The dotted red lines indicate the threshold provided by each web server to distinguish benign from pathogenic mutations. The values indicate the pathogenicity probability. (b) Evolutionary conservation of analyzed residues from 3L81 based on ConSurf. The region corresponding to the β‐sandwich subdomain B generally appears to be less conserved than subdomain A.

The pathogenicity of the selected set of protein variants was predicted using both the MutPred2 and E‐SNPs&GO web servers, while the conservation of wild‐type residues was examined through the ConSurf‐DB (Figure 4a,b) (Ben Chorin et al., 2020; Manfredi et al., 2022; Pejaver et al., 2020). Predicting the pathogenicity of patient‐related mutations might seem redundant. However, this approach can be insightful. Pathogenicity predictors offer scores that ideally reflect the severity of protein missense mutations. This is crucial when clinical information is unavailable or insufficient, as it often happens with RDs. Moreover, AI‐based tools in this study are trained on neutral versus pathogenic mutations from online archives and analyze target protein sequences in isolation. Comparing their output with clinical data reveals their reliability, particularly (A) for patient‐observed mutations and (B) for mutations affecting proteins in multimeric assemblies, such as the AP4M1 subunit.

Collectively, pathogenicity and conservation analyses revealed that the most pathogenic variants are E193K and Y284S, for which a consensus was reached, and the relative wild‐type residues are the most conserved too. C319R and C319Y immediately followed according to all three web servers. The most cryptic results were those regarding G307A. Overall, although being associated with SPG50, G307A was predicted to be the most benign according to both servers and, despite the low confidence, the least conserved as well. R227H was also labeled as probably not pathogenic, with a medium conservation score associated with the wild‐type residue. Although the predictions obtained for all SRVs showed overall coherence, the R367Q variant stood out for quite different computed pathogenicity scores by E‐SNPs&GO and MutPred2, though generally marked with a low pathogenicity likelihood. By means of ConSurf, we found out that R367 was assigned a medium‐to‐high conservation score, thus partially supporting the related pathogenic effect reported in the patient.

Notably, ConSurf also highlighted a specific conservation pattern across the AP4M1‐CTD (Figure 4b), with the structural portion corresponding to the β‐sandwich subdomain B (Burgos et al., 2010) being generally more variable than the rest of the CTD, suggesting that mutations occurring in this region might generally have less severe effects.

Subsequently, we sought to evaluate the structural profile of wild‐type residues at mutant sites to discriminate between mutations occurring at structured sites, and mutations falling in intrinsically disordered regions (IDRs). Since IDRs have widely been implicated in protein–protein interactions and in the mediation of conformational transitions (Latysheva et al., 2015), this step was necessary to understand whether our mutations could affect the binding capability and/or flexibility of our target. To this aim, we used the AIUPred web server (Erdős & Dosztányi, 2024). This tool provides a residue‐based score indicating the probability of each residue being inserted in structured versus disordered regions (Figure S1). We found out that all the missense variants from our dataset, but R367Q, fall in regions of AP4M1 with more structured profiles. This finding suggests that, while R367Q is expected to affect the capacity of AP4M1 to interact with other proteins, the remaining SRVs might rather impair the correct folding, thus leading to a loss of structural integrity.

Parallelly, we investigated solvent exposure of wild‐type residues at mutant sites, as disease‐related SRVs were shown to occur more frequently at buried residues (Savojardo et al., 2021). For this task, FreeSASA, a command line tool for calculating solvent‐accessible surface area, was used (Mitternacht, 2016). We took the relative solvent accessibility (RSA) as a measure of solvent exposure and, based on literature suggestions (Savojardo et al., 2021), we clustered our SRVs into two groups: those falling in solvent‐exposed protein regions (RSA >20%), and those located in buried portions (RSA <20%). All mutations but three (R227H, G307A, and R367Q) were found to affect buried amino acids, which turned out to be coherent with the pathogenicity scores obtained before. This finding strengthens the hypothesis that E193, Y284, and C319 might play a key role in ensuring the proper folding of AP4M1.

**FIGURE 5:**
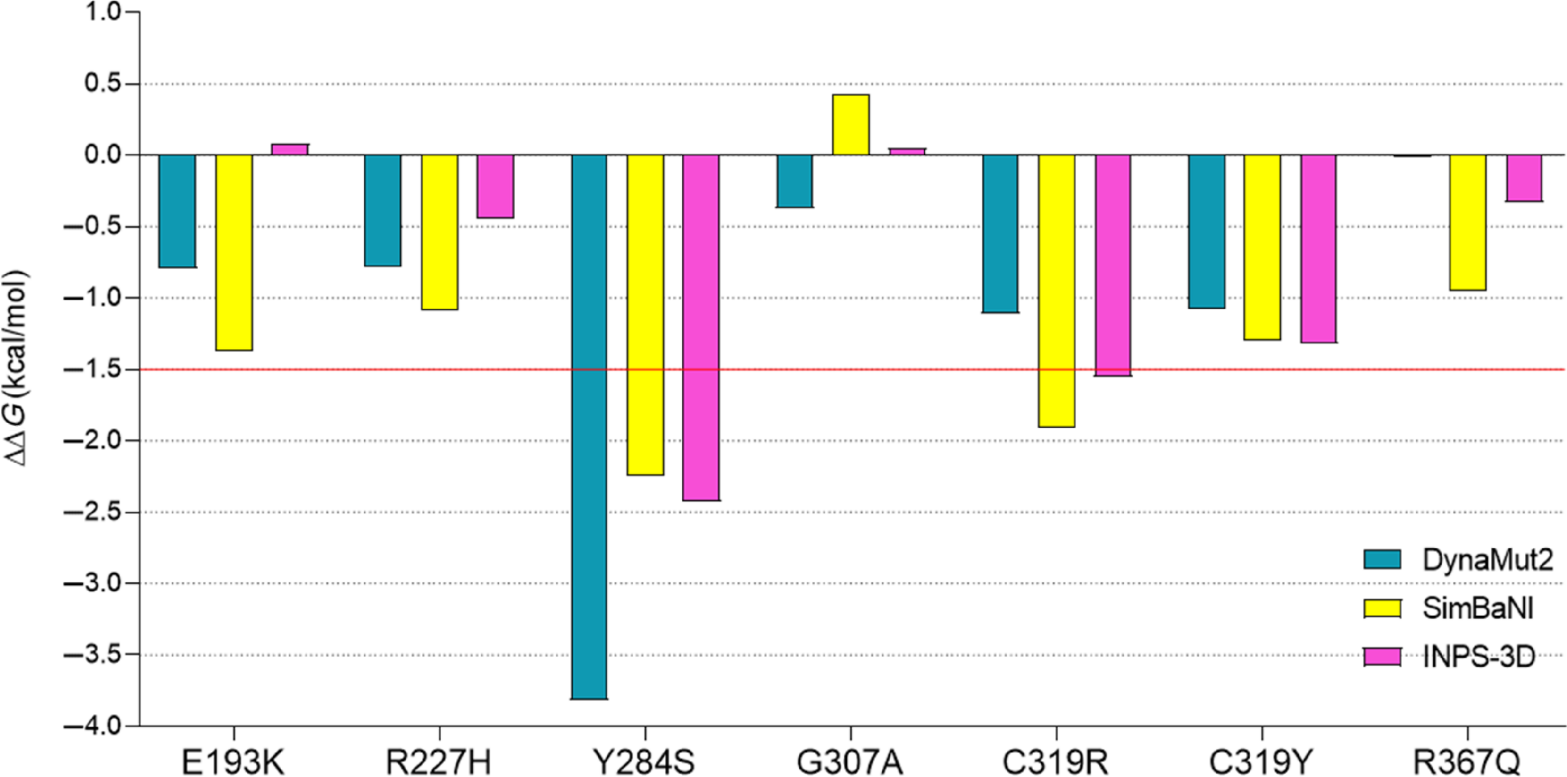
Effect of the different missense mutations on adapter protein complex 4 subunit mu‐1 (AP4M1) stability based on three different tools: DynaMut2 (blue), SimBaNI regression model (yellow), and INPS‐3D server (magenta). The cutoff at −1.5 kcal/mol is displayed as a red line.

The following step was focused on calculating the protein destabilization caused by each SRV. The more destabilizing a mutation is, the more it is likely to impact the proper folding of the target protein. This can result in a non‐functional product that may either be degraded or accumulated within cells, thus causing toxicity. Based on the previous findings, we expected a greater contribution to thermodynamic destabilization from mutations falling in structured and buried regions of AP4M1. To address this aspect, we used a consensus approach based on three different tools (DynaMut2, SimBaNI model, and INPS‐3D) (Caldararu et al., 2021; Rodrigues et al., 2021; Savojardo et al., 2016). All three tools provide a value of destabilization (expressed in kcal/mol as free energy variation) caused by missense mutations on a given protein. Based on previously published data, we fixed a cutoff of −1.5 kcal/mol to call highly destabilizing mutations (Bromberg & Rost, 2009; Gerasimavicius et al., 2020; Potapov et al., 2009; Seifi & Walter, 2018). From this analysis, Y284S and C319R were univocally elected as the most destabilizing mutations, with a destabilization exceeding the cutoff we fixed based on at least one tool (Figure 5). On the other hand, the impact of R367Q on protein stability was weaker, as previously postulated.

Table 2 summarizes the data collected so far to prioritize mutations eligible for the drug repurposing step. Variants positively selected during this stepwise evaluation encompass R227H, G307A, and R367Q. These three mutations met ligandability criteria, as they all were solvent‐exposed and not excessively structure‐destabilizing, and showed medium‐to‐high conservation scores and relatively low pathogenicity. We interpret the apparent paradox that some SPG50‐related mutations are predicted with low pathogenicity as a result of how prediction tools work, as anticipated at the beginning of this paragraph. Being from SPG50 patients, these mutations are actually pathogenic but the AP4M1 structural destabilization does not seem to be the only possible pathogenic mechanism taking place. Therefore, being these SRVs solvent‐exposed, we conclude that their pathogenic potential is due to impaired protein–protein interactions, which hardly emerge when the sequence of AP4M1 only is considered. It is relevant to underline that our analysis revealed how results from pathogenicity predictors must be critically assessed by means of complementary approaches, whenever possible.

**TABLE 2:** Summary of the results obtained from the various web servers and tools to establish the viability of adapter protein complex 4 subunit mu‐1 in the presence of each single residue variants from the addressed dataset.

Mutation	Tools																Ligandable?
	MutPred2		E‐SNPs&GO		ConSurf		AIUPred		FreeSASA		DynaMut2		SimBa‐NI		INPS‐3D		
E193K	Pathogenic	0.92	Pathogenic	0.93	Highly conserved	9	Structured	0.16	Buried	0.1	Destabilizing	−0.96	Destabilizing	−1.37	Stabilizing	0.17	⨯
R227H	Benign	0.40	Benign	0.27	Conserved	7	Structured	0.13	Exposed	33.7	Destabilizing	−1.28	Destabilizing	−1.09	Destabilizing	−0.59	✓
Y284S	Pathogenic	0.93	Pathogenic	0.95	Highly conserved	8	Structured	0.22	Buried	3.1	Destabilizing (over−threshold	−3.63	Destabilizing (over−threshold)	−2.25	Destabilizing (over−threshold	−2.27	⨯
G307A	Benign	0.48	Benign	0.02	Highly variable	1	Structured	0.17	Exposed	98	Destabilizing	−0.41	Stabilizing	0.43	Neutral	0.00	✓
C319R	Pathogenic	0.86	Benign	0.47	Conserved	7	Structured	0.15	Buried	0.4	Destabilizing	−0.77	Destabilizing, (over−threshold)	−1.91	Destabilizing	−1.53	⨯
C319Y	Pathogenic	0.89	Benign	0.42	Conserved	7	Structured	0.15	Buried	0.4	Destabilizing	−0.82	Destabilizing	−1.30	Destabilizing	−1.29	⨯
R367Q	Most probably pathogenic	0.61	Benign	0.05	Conserved	7	IDR	0.60	Exposed	57.2	Destabilizing	−0.26	Destabilizing	−0.95	Destabilizing	−0.35	✓

Abbreviation: IDR, intrinsically disordered region. Different colors highlight the overall take oninsights obtained from each of the tools employed.

### 2.4 Mutation‐related effects on inter‐subunit interactions in the AP‐4 core

Once that our SPG50‐associated SRVs were prioritized based on the properties listed in the previous section, we examined their putative roles within the AP‐4 core.

**FIGURE 6:**
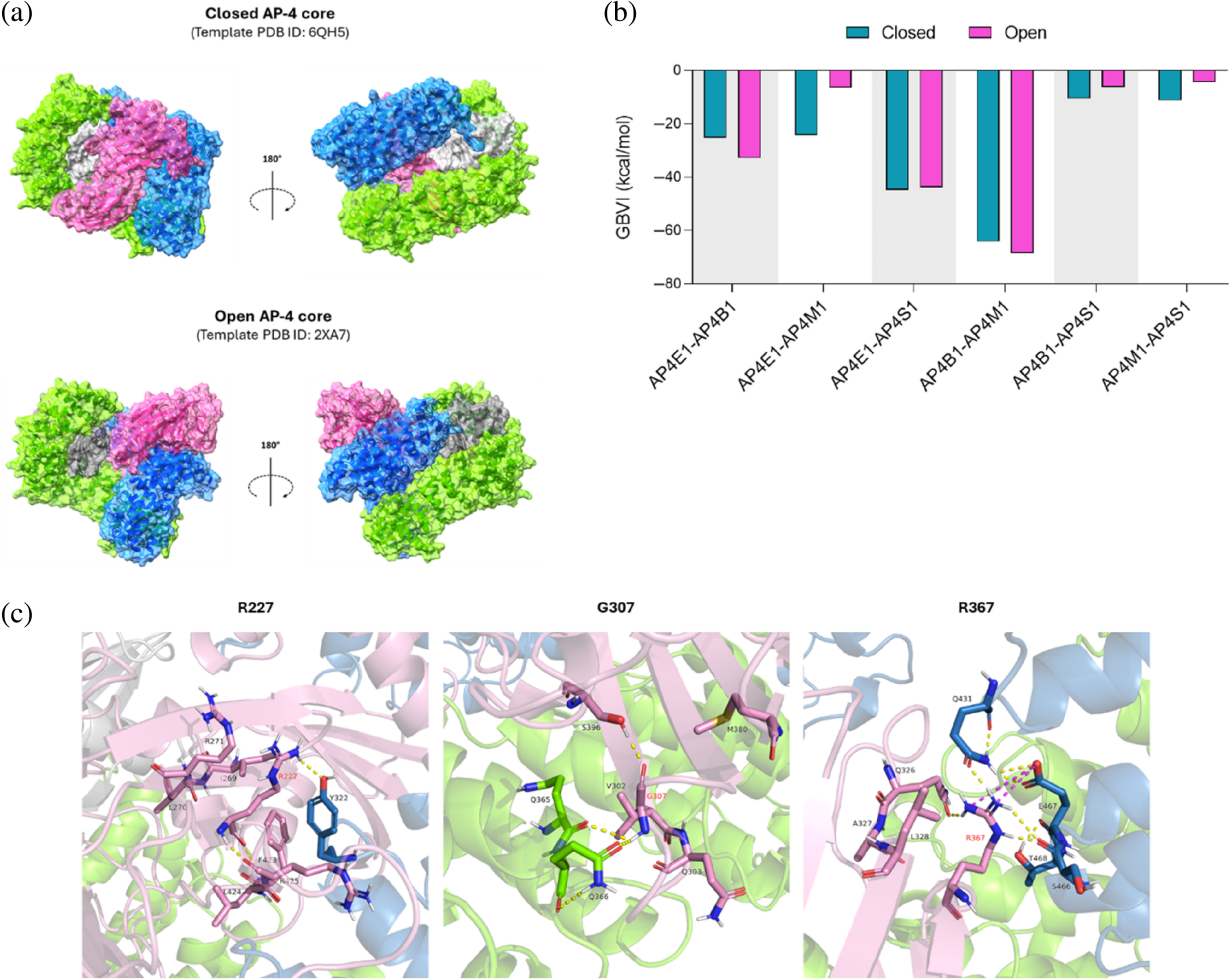
Homology models of the adapter protein complex 4 (AP‐4) core. (a) Closed and open AP‐4 cores generated from 6QH5 and 2XA7 crystals of the AP‐2 core, respectively (blue: AP4B1; green: AP4E1; pink: AP4M1; gray: AP4S1). (b) Inter‐subunit total binding affinity (GBVI) values indicate a stronger association (i.e., more negative values) within the AP4B1‐AP4M1 and AP4E1‐AP4S1 hemicomplexes overall, both in the closed and open conformations (blue: Closed AP‐4 core; magenta: Open AP‐4 core). (c) Interactions established by wild‐type R227, G307, and R367 at protein–protein interfaces in the closed AP‐4 core (yellow dotted lines represent hydrogen bonds, while salt bridges are displayed in magenta). AP4B1, adapter protein complex subunit beta‐1; AP4E1, adapter protein complex subunit epsilon; AP4M1, adapter protein complex 4 subunit mu‐1; AP4S1, adapter protein complex subunit sigma‐1.

As anticipated in the Introduction, no experimental structure of the AP‐4 heterotetramer is available. However, crystal structures of the core domain of the AP‐1 and AP‐2 complexes have been deposited on the RCSB Protein Data Bank over the years. Therefore, we selected two AP‐2 crystals, 6QH5 and 2XA7, to serve as templates for modeling the closed and open states of AP‐4 core, respectively (Figure 6a). The selection of templates aligned with the rationale provided by Gadbery and colleagues (Gadbery et al., 2020). The two homology models of the AP‐4 core domain were built using the SWISS‐MODEL server (more details are discussed in Section 4), and inter‐subunit binding affinity values were calculated in both states. The strongest interactions were highlighted for the AP4B1‐AP4M1 and AP4E1‐AP4S1 hemicomplexes in both conformations (Figure 6b). This finding is in line with the hypothesized assembly model (Mattera et al., 2022), where these two hemicomplexes are stabilized separately first, and then combined in the final tetrameric structure.

According to our AP‐4 models, R227, G307, and R367 are all involved in inter‐subunit contacts in the closed AP‐4 conformation (Figure 6c). In particular, R367 was found to establish an ionic bond with residue E467 from AP4B1. Remarkably, this bond was associated with a high interaction energy (Table S2). The fact that these three mutations occur at protein–protein interfaces might justify the lower pathogenicity detected by MutPred2 and E‐SNPs&GO, which took into account the AP4M1 sequence rather than the AP‐4 tetramer.

**FIGURE 7:**
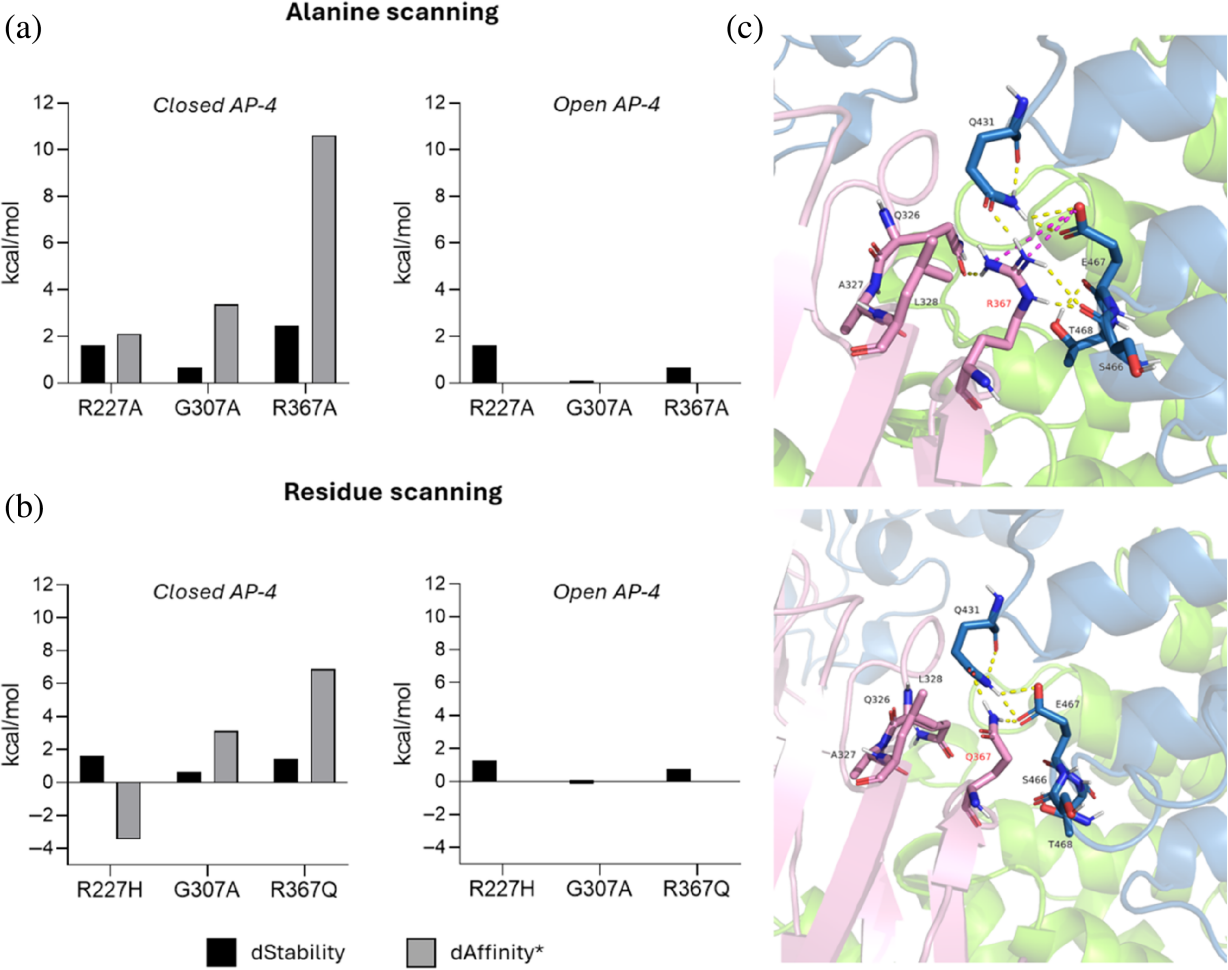
Single residue variants‐based interaction energy profile (positive values: Loss of stability and/or affinity). (a) Alanine scanning results from MOE show that the most serious impact on protein stability and affinity is highlighted downstream of R367Q in the closed adapter protein complex 4 (AP‐4) core model, as the associated energy variations are increased. (b) Residue scanning analysis shows a similar impact on both stability and affinity with respect to alanine scanning with respect to R367Q in the closed AP‐4. A stabilizing effect emerged for R227H in the closed tetramer, although no clear explanation from direct visualization did emerge (Figure S2). (c) Wild‐type versus mutant interaction scenario of R367Q in the closed AP‐4 core: The substitution of the arginine 367 with glutamine leads to the abolition of a critical salt bridge with adapter protein complex subunit beta‐1 (AP4B1)‐E467, thus explaining the impact on the target properties highlighted by means of both alanine and residue scanning. *dAffinity was calculated with respect to the AP4B1 chain for both R227H and R367Q, while to adapter protein complex subunit epsilon for G307A.

At this point, we wondered whether the corresponding SPG50‐associated mutations could impair the integrity of the AP‐4 core. To this aim, we adopted two different approaches. In the first one, we exploited the Protein Design application integrated within the MOE suite. We submitted our mutations to the alanine scanning tool (Figure 7a), which aims at determining how specific residues impact on protein properties (including stability and affinity) by mutating them to alanine, which side chain is “inert” (nonpolar and with minimal steric hinderance). Parallelly, we exploited the residue scanning tool to specifically assess the effect of our missense mutations on stability and affinity of the AP‐4 core homology model (Figure 7b). Both analyses highlighted that R227H, G307A, and R367Q all affect the integrity of the closed AP‐4 core rather than that of the open core. This is consistent with prior observations, as all wild‐type residues are involved in inter‐subunit bonds in the closed AP‐4 state.

As for the second approach, we compared the interactions established by the considered AP4M1 residues (i.e., positions 227, 307, and 367) in wild‐type and mutant core models (Figure S2). Results suggest that R227H, in the closed conformation, leads to the loss of a hydrogen bond with residue Y322 on the AP4B1 subunit while, in the open AP‐4 state, this mutation disrupts the ionic bond with residue E263 from AP4M1. In the latter case, the disruption of this ionic contact seems to increase the distance between the α‐helix containing E263 and the β‐sheet region formed by the remaining interacting residues. As regards G307A, this variant is characterized by the loss of a hydrogen bond with Q366 on the AP4E1 subunit in the closed core, while in the open state no significant changes emerge. Finally, R367Q in the closed core is responsible for the loss of a salt bridge with the E467 residue of AP4B1 (Figure 7c), while one less hydrogen bond with residue N329 of AP4M1 was found in the open conformation.

The greatest impact on stability and affinity was highlighted for R367Q in the closed AP‐4 core, and we hypothesized that this arginine could be an interface hot spot residue (David & Sternberg, 2015). Hot spots at protein–protein interfaces are key residues that address the majority of the interaction between two protein entities, and their mutation to an inert amino acid (e.g., alanine) leads to a ≥2 kcal/mol reduction in binding free energy (Clackson & Wells, 1995; David & Sternberg, 2015; Keskin et al., 2005). The impact of such a mutation on the integrity of the tetramer is further justified by the fact that the adapter protein complex 4 might follow a step‐wise assembly model, where the AP4B1‐AP4M1 hemicomplex represents one of the two building blocks the final assembly starts from. Therefore, the integrity of this first hemicomplex is paramount to ensure the stability of the whole complex as well, as discussed above. Furthermore, R367 belongs to an electropositive protein patch that, based on homology with AP‐1 and AP‐2, could be involved in the interaction with negatively charged phosphoinositides on membranes to mediate the complex recruitment to target organelles (Figure S3) (Canagarajah et al., 2013; Collins et al., 2002). This electropositive patch on AP4M1, which includes R367, is coplanar to a bigger one on the AP4E1 subunit when the AP‐4 complex is in the open state (i.e., the conformation allowing membrane binding and cargo recognition). This is coherent with the highlighted interaction modality between homologous subunits in AP‐2 and membranes to mediate the recruitment of the complex and cargo loading at target organelles (Canagarajah et al., 2013).

### 2.5 Binding pocket search

In the previous section, we predicted an impact on the stability of the AP‐4 core downstream of R227H, G307A, and R367Q owing to the weakening of PPIs. Therefore, we envisioned a potential therapeutic mechanism by any molecule able to restore the correct affinity between the AP‐4 subunits. It follows that the next step of the study was to look for a pocket at the PPIs containing these mutations, aiming at finding compounds capable of “bridging” the subunits back together again.

**FIGURE 8:**
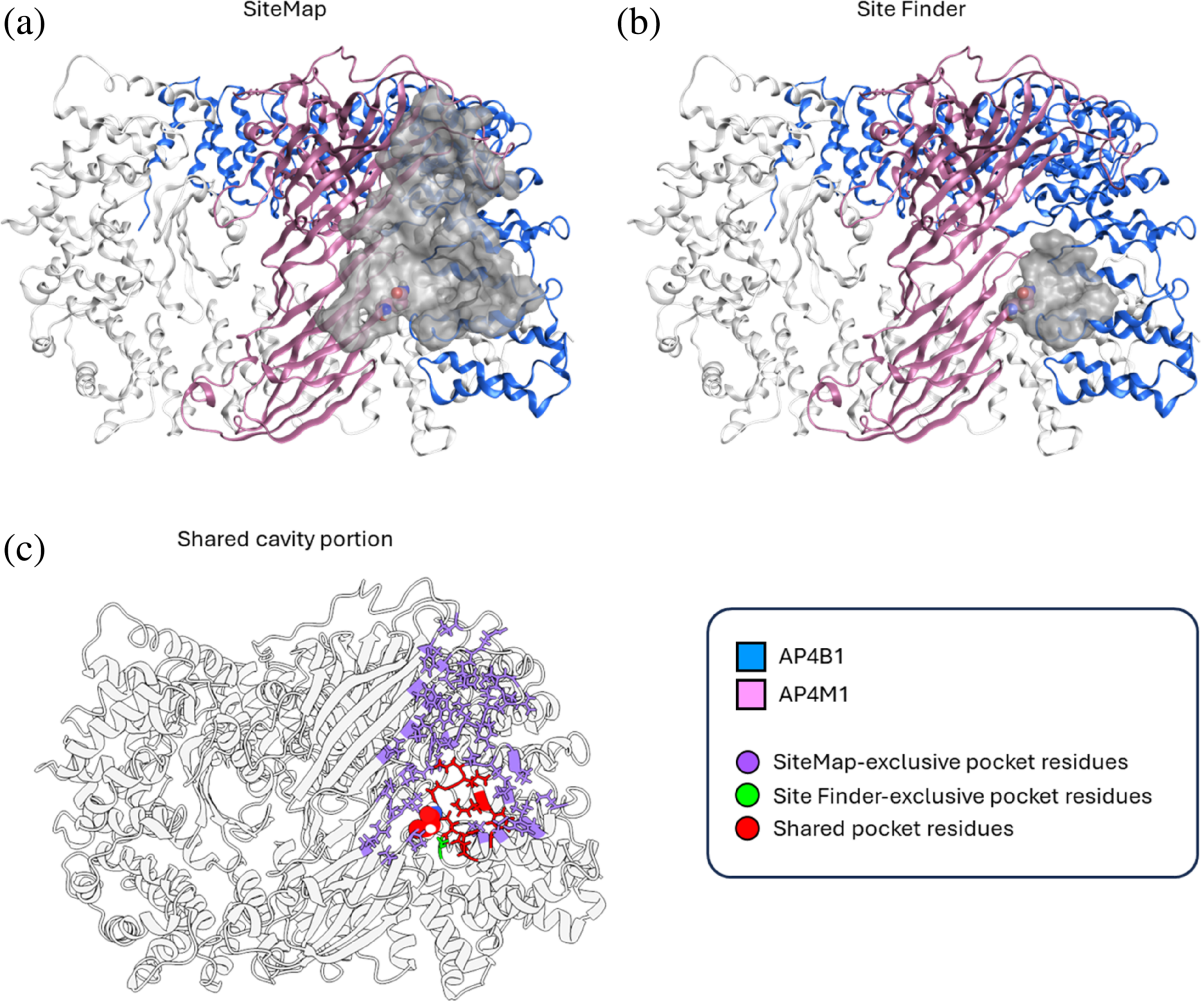
Binding pocket definition based on Schrödinger SiteMap (a) and MOE‐SiteFinder (b). The pocket surface is displayed in gray, with the Q367 residue shown as spheres. (c) Highlight on residues encompassed by the SiteMap pocket (purple), by the Site Finder cavity (green) and on those common to both pockets (red). Q367 falls in the common area and is displayed as spheres. From this figure, it is possible to appreciate that the Site Finder cavity is almost entirely included within the SiteMap pocket, with the exception of adapter protein complex subunit beta‐1 (AP4B1)‐S466. AP4M1, adapter protein complex 4 subunit mu‐1.

A pocket search was performed using two commercial tools to reach a consensus (SiteMap (Halgren, 2007), MOE‐SiteFinder), as well as two free web servers (DoGSiteScorer, FPocketWeb) (Kochnev & Durrant, 2022; Volkamer et al., 2012) to further test the outcome of the two previous algorithms (data not shown). Most solid results, in terms of scores, were obtained for the closed R67Q mutant AP‐4 core, where a pocket including Q367 and at the interface between AP4B1 and AP4M1 was robustly highlighted (Figure 8). Of note, unbiased search with SiteMap first highlighted a larger binding cavity (Figure 8a), while a search focused on Q367 with MOE‐SiteFinder identified a smaller site (Figure 8b), almost entirely comprised within the first one (Figure 8c). On the other hand, inconsistent data emerged both for the closed R227H‐AP‐4 and G307A‐AP‐4 mutants. According to these and previous results, we focused drug repurposing efforts on the R367Q variant only.

### 2.6 Drug repurposing for R367Q

**FIGURE 9:**
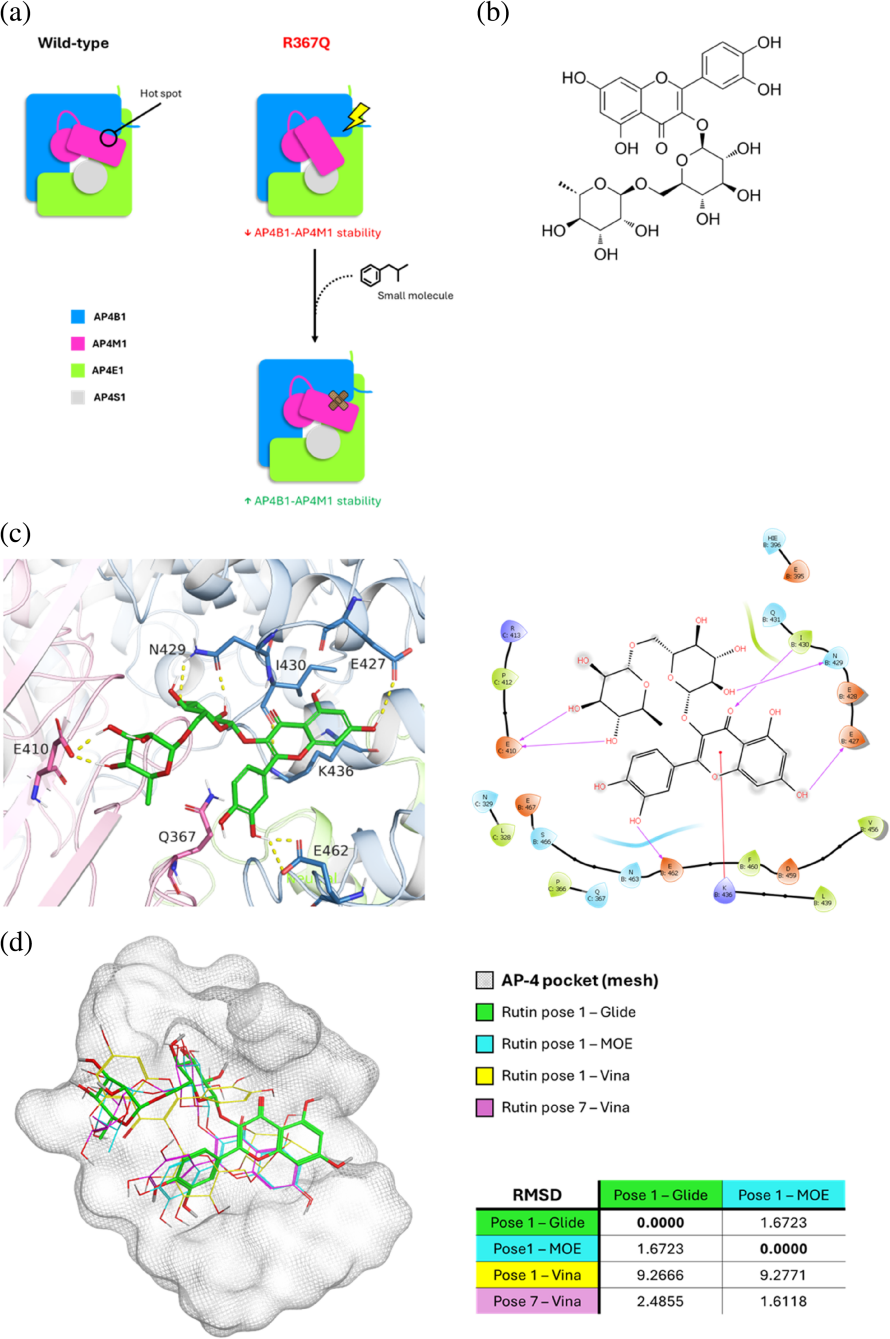
Virtual drug screening on the selected adapter protein complex 4 (AP‐4) pocket. (a) Proposed mode of action of a small molecule to restore the binding affinity between subunits adapter protein complex subunit beta‐1 (AP4B1) and adapter protein complex 4 subunit mu‐1 (AP4M1). (b) Structure of rutin, a flavonoid compound consisting of a quercetin with the hydroxy group at C‐3 substituted with the disaccharide rutinose (molecular weight: 610.52 g/mol). (c) Best docking pose of rutin according to Glide shown together with AP4B1 and AP4M1 residues involved in the interaction. The interaction diagram shows that rutin is able to establish hydrogen bonds with residues E427, N429, and I430 from AP4B1, as well as with E410 from AP4M1. A cation‐pi interaction is also formed with AP4B1‐K436. (d) Comparison between binding modes of rutin according to Glide (in green), MOE‐Dock (cyan), and Vina (yellow and magenta). RMSD values (in Å) indicate that Glide and MOE‐Dock best poses of rutin are more similar with respect to the first pose highlighted by AutoDock‐Vina. However, pose 7 by Vina displays remarkable superposition with pose 1 from MOE‐Dock. Bold indicates the matrix diagonal.

The idea behind drug repurposing of the SPG50‐associated, R367Q variant was to rescue the loss of affinity between subunits AP4B1 and AP4M1 at the critical spot where Q367 falls. Therefore, we wanted to look for compounds with putative glue mode of action (MoA) to restore the interaction between these two subunits (Figure 9a). To do this, we performed a virtual screening of a library of compounds from the DrugBank database (Knox et al., 2024). Molecule selection was based on the drug group (approved and nutraceutical), as well as on chemical considerations (see Section 4).

Virtual screening was first performed using the open‐source AutoDock‐Vina software (Trott & Olson, 2010). From this initial experiment, the flavonoid rutin turned out to favorably bind to our target with an affinity of −7.89 kcal/mol (see Section 4), while being a natural product, previously reported to be a dietary antioxidant with a neuroprotective effect (Habtemariam, 2016; Sun et al., 2021; Xu et al., 2014). Rutin is made of a rutinose disaccharide group bound to a quercetin unit (Figure 9b). We decided to validate the first screening with two commercial docking software: Glide (Halgren, 2007) and MOE‐Dock. The same dataset of compounds was submitted to both gold standard tools, and once again rutin emerged as a promising candidate with scores of −9.72 and −4.34 (Glide docking score and MOE S score, respectively; see Section 4 for more details). We repeated the docking of rutin at a higher accuracy level with Glide and identified its best binding pose within the AP‐4 pocket. According to this pose, Q367 is not directly involved in the binding to rutin (Figure 9c). Nevertheless, we did not consider this as a huge problem, as rutin still manages to bind in colocalization with Q367 at the investigated spot. Repetition of docking with MOE‐Dock revealed overall coherence with Glide, with the quercetin and rutinose groups similarly oriented in the cavity (Figure 9d). However, slight differences in the docking poses led to differences in the interacting residues (Figure S4). We interpret this outcome as due to the different force fields employed by the docking engines, as well as to different receptor preparation methods. Finally, accurate docking was repeated with AutoDock‐Vina as well, although more remarkable differences in the binding pose emerged this time compared to the previous tools.

Altogether, these results highlighted a candidate drug, rutin, expected to bind residues from both AP4B1 and AP4M1 at the interface spot, where an R367Q‐dependent destabilization is predicted to occur. Thus, these data support a glue MoA mediated by this small molecule.

## 3 DISCUSSION

Molecular modeling pipelines, made of both web servers and standalone programs, harness significant potential for drug discovery and for the elucidation of molecular mechanisms underlying RDs. This study focuses on the SPG50/AP4M1 pair and reports a new computational strategy testing the feasibility of personalized computational medicine and suggesting treatment options.

To achieve our objectives, we employed two new tools. The first one, DRARDT, is a method demonstrating that a thorough evaluation of the target—including aspects like structural context and interactome—is essential to any computational approach aimed at discovering a drug to modulate that target. The second tool, 3DVarPro, is a novel visualization platform through which we conducted a qualitative analysis of the overall structural context of SPG50‐associated missense mutations from ClinVar. This analysis revealed that these mutations are uniformly distributed throughout the AP4M1 structure. Results provided by DRARDT and 3DVarPro support that designing and implementing new, ad hoc systems for computer‐aided drug discovery are nowadays pivotal to foster efforts in this field of research.

Another key step of this study is represented by the identification and prioritization of a small set of SRVs eligible for personalized drug discovery efforts. This was achieved by using a variety of tools to select mutants that are less likely to undergo NMD, premature protein degradation or unfolding. This is particularly important for our approach, which relies on the availability of the target protein within cells.

We modeled the structure of the AP‐4 core in both closed and open states. This was necessary to suggest pathological mechanisms for different mutations: three of these variants (R227H, G307A, R367Q) are located at protein–protein interfaces, indicating a potential loss of affinity between AP‐4 subunits at critical hotspot regions when mutation occurs. The strongest destabilization of the AP‐4 complex was predicted for R367Q due to the loss of a critical salt bridge. A consequence of this finding is that a potential mechanism to counteract the mutation effect can be a small‐molecule drug able to bind both AP‐4 subunits, thus restoring their reciprocal affinity. This MoA is known in medicinal chemistry by the name of molecular glue (Domostegui et al., 2022) and supports, at least in principle, the druggability of the R367Q mutation.

Ligandability (i.e., the potential to be bound by a small molecule) is a key requirement to ensure the druggability of a protein target (Di Palma et al., 2023). This property is conditional to the presence of a binding cavity within the desired receptor site. To address this point, we screened our AP‐4 core models for putative binding cavities including any of the mutant residues and found out that only R367Q was included in a binding pocket with sufficient confidence. However, in this study, we did not account for transient pockets on the protein surface. This should be considered in the future.

Finally, we performed virtual screening using three different docking engines, as it is our opinion that a consensus approach is advisable for more solid results. All three docking tools pointed out the flavonoid rutin as a candidate molecule to rescue the interaction between AP4B1 and AP4M1. Although not directly binding to the mutant residue, rutin was found to establish contacts with surrounding residues from both AP4M1 and AP4B1. These findings support rutin as a drug candidate for the R367Q variant, but open to the question of whether variants in the same position could benefit from this treatment too.

To obtain proof‐of‐concept results, patient‐derived cells such as skin fibroblasts represent an opportunity (Rossi Sebastiano et al., 2024). This perspective is especially viable considering that established cell markers are already available to monitor the effect of AP‐4‐related HSP forms on cell models (Ebrahimi‐Fakhari et al., 2021). Thus, further computational and experimental efforts are needed to support the validity of the suggested mechanism, the efficacy of rutin and its applicability to tackle other mutations as well.

## 4 MATERIALS AND METHODS

### 4.1 3D structures

Structures used in this study include the experimental AP4M1 crystal (PDB 3L81; Accessed July 2023), its predicted structure available in the AlphaFold database (ID AF‐O00189‐F1; Accessed July 2024), and two crystals of the AP‐2 core (PDB IDs 6QH5 and 2XA7; Accessed November 2023). From the AlphaFold database, data from the AlphaMissense prediction model relative to AP4M1 were downloaded as well to produce the color map at Figure 2b (Accessed June 2024). The AlphaMissense model predicts a pathogenicity score ranging from 0 to 1 for each of the possible 19 amino acids substitutions and the color coding of the 3D structure is proportional to average values from all possible substitutions.

### 4.2 3DVarPro web app

The 3DVarPro web app was developed in Python language through the Streamlit framework. The user is required to provide a gene name or UniProt identifier as input. Optionally, a PDB file of the target from the Protein Data Bank can be uploaded too (differently, 3DVarPro can automatically retrieve and upload the AlphaFold prediction of the target). The current version (1.2.1) does not support PDB files containing more than one model, more than one chain, or discontinuous chains. The user is free to custom visualization options by choosing associated traits, mutation position, B‐factor/pLDDT threshold, protein color and style, and both color and style of mutation sites. Associated traits taken into account in this study included (1) AP‐4 deficiency syndrome, (2) HSP 50, (3) HSP, (4) spastic tetraparesis, and (5) spastic paraplegia. Zooming on specific residues is also permitted. 3DVarPro is available at https://3dvarpro.streamlit.app/, while a public release of the code can be found at https://github.com/atturk/3DVarPro.

### 4.3 DRARDT method

Literature research was performed by using the advanced search section of the PubMed archive to look for publications including the text word “AP4M1” from 2000 to 2024 (Accessed March 11, 2024). The definition of the score thresholds reported in Figure 2 is based on an internal study of published and unpublished RD‐causing mutations. Thresholds were tentatively established based on such analysis by considering the distribution of literature abundance, and it will be subjected to future revision when suggested by the application of the method to other systems. Anyway, they currently provide a reliable estimation of the amount of the information available in the literature.

NMD susceptibility was predicted for nonsense and missense mutations with the MutationTaster2021 web server by fetching it with gene symbol (AP4M1), reference transcript (ENST00000359593), the position of the single nucleotide variant with respect to the coding sequence (c.), and the new base. The NMDEsc Predictor server was used to predict NMD likelihood for frameshift variants by providing the RefSeq transcript ID of the target (NM_004722), coding position at frameshift mutation (c.), and frameshift effect (e.g., +1 for c.218dupA). Both resources were accessed on May 25, 2023. Finally, the results of this analysis are summarized by calculating the percentage of mutations leading to NMD by applying a consensus approach. Thresholds were established based on the distribution of literature cases.

Structures 3L81 and AF‐O00189‐F1 were visualized in the UCSF Chimera environment (v1.17.3). Residues falling in a 5 Å‐radius‐zone from each NMD‐negative residue undergoing mutation were analyzed in terms of B‐factor (3L81) or pLDDT (AF‐O00189‐F1) and classified in three classes based on specific thresholds: High, Medium, and Low. The thresholds for the temperature factor of experimental 3D structures were assigned as follows: Bfactor <30 Å^2^ (High), 30 < Bfactor <60 Å^2^ (Medium), and Bfactor >60 Å^2^ (Low). Similarly, but with another distribution, thresholds were so established for pLDDT: pLDDT <50 (Low), 50 < pLDDT <70 (Medium), and pLDDT >70 (High). This choice is rooted in the fixed (0–100) values and on what was suggested by Jumper and colleagues in the original AlphaFold2 publication (Jumper et al., 2021). Out of 61 residues surrounding the analyzed mutation sites, 51 were labeled as “High” and 10 as “Medium” in both experimental and predicted structures. In both cases, the final value was assigned by considering, for each residue under exam, the average of the surrounding amino acids. Both the temperature factor and the pLDDT are considered as measures of the flexibility of the considered protein region, as well as uncertainty in the position of atoms in the vicinity of the mutation. In principle, a high degree of flexibility and uncertainty is reflected in poor modeling of the structure around the mutation and thus in lower reliability of predictions. High values indicate more stable and reliable contexts.

The interactome of AP4M1 was investigated by addressing the STRING‐DB (https://string-db.org/) and KEGG pathway maps (https://www.genome.jp/kegg/pathway.html) (Accessed October 2023). The STRING‐DB was fetched with the target name and Homo sapiens as organism. Settings were modified to display data from *Experiments* and *Databases* only. Interactors with confidence lower than medium were ignored. In this case, indexes were assigned using a miscellaneous approach: for <3 interactors in the STRING‐DB, a “Low” flag is assigned, while in the same case but with the protein being at least in one cellular pathway from the KEGG database, the “Medium” label is chosen instead; finally, when the number of STRING interactors was >3, a “High” flag is assigned. Also in this case, DRARDT was validated on a set of mutations associated with different types of paraplegia that were confidentially reported from third party collaborators.

### 4.4 Collection of pathogenicity and conservation data

Pathogenicity of selected variants was predicted using both the MutPred2 and E‐SNPs&GO web servers (Accessed October 2023) (Manfredi et al., 2022; Pejaver et al., 2020). Both tools require the protein primary sequence and a list of amino acid substitutions as input. They both rely upon machine learning models to provide a non‐energy‐based prediction of pathogenicity for each given substitution. The output from E‐SNPs&GO also comes with a reliability index ranging from 0 (minimum confidence) to 10 (maximum confidence) (Manfredi et al., 2022). E‐SNPs&GO is based on protein language models and focuses on the protein sequence and related functions by extracting Gene Ontology (GO) functional annotations (Manfredi et al., 2022). MutPred2 relies upon both genetic and molecular data (e.g., conservation and substitution likelihood of single residues, and structural features of the target) (Pejaver et al., 2020). Both tools were addressed in order to reach a consensus prediction for our set of SRVs. For MutPred2 predictions, a threshold of 0.80 was considered based on the guidelines at the web server to reduce the false‐positive rate (although probabilities greater than 0.50 should theoretically indicate pathogenicity). As for E‐SNPs&GO outputs, variants predicted to be pathogenic were those with *p* ≥ 0.50, although their reliability index was taken into account as well. Parallelly, the evolutionary conservation of AP4M1 residues undergoing mutation was assessed using the ConSurf‐DB (query: 3L81 crystal. Accessed October 2023) (Ben Chorin et al., 2020). ConSurf works by looking for homologous sequences with respect to the query to then generate a multiple sequence alignment (MSA), and ultimately calculate conservation scores for each residue of the query (Glaser et al., 2003). In particular, we focused on the 3L81 structure of AP4M1 (Figure 3b), since our mutations all fall in the MHD.

### 4.5 Flexibility, RSA, and thermodynamics

Disordered AP4M1 regions were identified using the AIUPred web server (Accessed May 2024) by fetching the UniProt ID of AP4M1 (primary sequence) and using default parameters. Higher scores correlate to a higher probability of disorder. We called disordered versus structured regions by considering a cutoff of 0.50 as reported in the relative original publication (Dosztányi, 2018).

The RSA values of residues from AP4M1 were calculated using FreeSASA (v2.1.2) in Ubuntu 20.04 LTS from the AlphaFold structure of the target. Used settings were solvent radius = 1.4 and input = naccess. RSA is calculated as follows:

$$\mathrm{RSA}=\text{SASA}/{\text{SASA}}_{\max}$$

where ${\text{SASA}}_{\max}$ (maximum solvent‐accessible surface area) is calculated on an Ala‐X‐Ala tripeptide (X = residue the RSA is being calculated for) because of the minimal steric hindrance of alanine. Both experimental and predicted structures of AP4M1 were submitted to this tool, though the results obtained in both cases were comparable.

The mutation‐related effects on AP4M1 stability (∆∆G) downstream of missense variants were obtained starting from the 3L81 crystal of the target, together with the list of missense mutations, by using a consensus approach based on the DynaMut2 (Rodrigues et al., 2021) and INPS‐3D (Savojardo et al., 2016) web servers (https://biosig.lab.uq.edu.au/dynamut2/ and https://inpsmd.biocomp.unibo.it/inpsSuite/default/index3D, respectively; Accessed November 2023), as well as the SimBaNI model described by Caldararu and co‐workers (Caldararu et al., 2021). DynaMut2 is an online tool that, starting from the 3D structure of a protein, applies Normal Mode Analysis to study protein movements and graph‐based methods to evaluate the native protein environment to predict the effect of SRVs on protein stability and dynamics. INPS‐3D also starts from a 3D structure, and it extracts descriptors such as the RSA of wild‐type residue, the local energy difference between wild‐type and mutant residue, and other sequence‐based descriptors. Finally, SimBaNI is a multilinear regression model that does not require any structure as input but considers three distinct molecular properties (solvent accessibility of wild‐type site, volume difference, and polarity difference upon mutation) to calculate the effect of SRVs on protein stability.

### 4.6 AP‐4 models' generation and evaluation

Homology modeling of the wild‐type closed and open AP‐4 cores was performed using SWISS‐MODEL (https://swissmodel.expasy.org/. Accessed June 5, 2023) starting from the 6QH5 and 2XA7 crystals, respectively. In both cases, AP‐4 subunits were modeled starting from their primary sequences provided in FASTA format and downloaded from UniProt (AP4B1: Q9Y6B7; AP4M1: O00189; AP4E1: Q9UPM8; AP4S1: Q9Y587). Only the core domain of AP‐4 was generated since all mutations taken into account fall in this domain. We also evaluated the homology between the employed AP‐2 subunits in the templates and the primary sequences of the AP‐4 subunits. Although the degree of homology between AP‐2 and AP‐4 subunits is lower compared to AP‐1 and AP‐2 subunits (data not shown), we considered our homology models valid for the purpose of our analysis due to the high structural conservation across all eight monomers, and expected AP‐4 to assemble in the same fashion as AP‐1 and AP‐2.

The resulting models were subsequently mutated to generate the R227H, G307A, and R367Q mutant models using the Dunbrack rotamer library implement in Chimera (v1.17.3) (backbone: fixed). Wild‐type and mutant structures were then prepared and minimized via the Molecular Operating Environment (MOE 2022.2): in the Structure Preparation panel, models were first corrected for issues, then protonated using the Protonate3D application (default settings but pH set to 7.4), and finally minimized through the Energy Minimize panel (forcefield: Amber10:EHT; RMS gradient: 0.01 kcal/mol/Å^2^; planar systems treated as rigid bodies. Remaining options left as from default). In wild‐type models, inter‐subunit binding affinities and residue‐based contacts from Table S2 were calculated from the Protein > Contacts application within MOE by testing one subunit against the other in the former case and selecting residues undergoing mutation against all those from the interacting subunit in the latter. Residues undergoing mutation in wild‐type AP‐4 core models were also submitted to the Protein Design tools, alanine scanning and residue scanning, to predict the effect of SRVs on stability and affinity (parameters: Amber10:EHT force field and R‐field 1:80. Rest as from default). Electrostatic potential surface map of both wild‐type and R367Q open AP‐4 cores was computed in ChimeraX (v1.7.1).

### 4.7 Pocket screening

MOE‐SiteFinder was used on both closed and open AP‐4 core models by centering the search on H227, A307, and Q367, and defining a radius of about 10 Å. The propensity for ligand binding index was chosen as metric to evaluate the identified cavities. Pockets with propensity for ligand binding <0.00 were discarded. Only Q367 in the closed AP‐4 model passed this selection. Schrödinger SiteMap was used on the whole AP‐4 core models (re‐prepared in Maestro via the Protein Preparation Wizard) with default parameters and reporting up to 15 sites, including shallow binding sites. A pocket including Q367 with SiteScore 0.960 and Dscore 1.088 was highlighted in the closed AP‐4 mutant model, but none including neither H227 nor A307. Finally, a pocket including Q367 in the closed AP‐4 core was also detected by DoGSiteScorer (https://proteins.plus/) and FPocketWeb v1.0.1 (https://durrantlab.pitt.edu/fpocketweb/) with default settings (Accessed June 2024) (data not shown). We screened all three closed AP‐4 mutant core models for pockets including any of the three residues undergoing mutation, as well as the open AP‐4^R227H^ core for a pocket including H227, since a more significant loss of affinity and/or structural stability was observed in this scenario, and further justified by the observation of the loss of a critical ionic bond (Figure S4). This latter case is the only one where protein–protein interactions are not affected, but rather a destabilization within the AP4M1 subunit is likely occurring.

### 4.8 Structure‐based virtual screening

Compounds for virtual screening were downloaded as 3D‐SDF from DrugBank (v5.1.12) and then filtered, based on the number of rotatable bonds (<13) and molecular weight (100–1000 Da), as both accuracy and speed of docking engines are generally lower for large and highly flexible molecules (Dhanik et al., 2013; Guo et al., 2014). Moreover, heavy molecules are less likely to cross the blood–brain barrier, while too small ones were not suitable for the relatively large pocket they were screened against. Ligands were further filtered after virtual screening based on their indication, meaning that compounds with nonnegligible side effects and nonoral routes of administration (for which patient compliance is lower) were ignored (e.g., chemotherapeutics, contrast agents, etc.) to consider those with acceptable therapeutic index only. Resulting molecules were then prepared and standardized using an in‐house protocol combining RDKit and MOE, to ensure correct chemical representation and the major ionization specie at physiological pH. Finally, the Python Meeko package was employed for conversion to PDBQT format (required by AutoDock Vina).

AutoDockTools1.5.7 was used to prepare the R367Q mutant AP‐4 core in closed state for virtual screening with AutoDock‐Vina (v1.2.3): hydrogens were corrected and Kollman charges added.

AutoDock‐Vina was run in batch mode with the following parameters: exhaustiveness 16, number of modes 10, and energy range 3. The docking box was centered on Q367 (coordinates: −51.5, −19.5, 281.7), with size along coordinates set to 25, 20, and 30 Å respectively. Output affinities (kcal/mol) ranged from −14.54 (highest affinity) to −2.91 (weakest interaction).

Prior to virtual screening with Glide (via Virtual Screening Workflow in Maestro v12.5), the drug library was prepared with LigPrep (OPLS3e force field, protonation states at pH 7.0 +/− 2.0 generated with Epik, desalting and tautomer generation set on, chirality determined from 3D structure), and the receptor grid generated via the Receptor Grid Generation panel (Q367 picked as the center of the box, with size along center coordinates set to 25, 20, and 30 Å as for Vina). Glide virtual screening was launched from the Virtual Screening Workflow panel. All virtual screening steps (HTVS, SP, XP) were allowed to dock flexibly, to perform post‐docking minimization, and to keep 50% of best compounds from each step (HTVS stage: all states retained; SP stage: all good scoring states retained; XP: only best scoring states retained). Glide docking scores ranged from −17.28 (best outcome) to 1.59.

For virtual screening with MOE‐Dock, the docking box corresponded to the pocket highlighted with SiteFinder, while the drug library was washed for correct protonation and desalting and minimized with RMS gradient set to 0.1 (MMFF94x force field) to obtain starting conformers. The triangle matcher placement method was used to generate up to 30 poses, refinement set to none to speed up the process, and affinity‐dG selected as scoring function. The final scores (*S*) ranged from −6.27 (best) to 9.90 (worst).

Docking of rutin only to identify the most favored binding pose was performed with AutoDock‐Vina at exhaustiveness 500. Glide was used at extra precision (XP) with the same aim. MOE‐Dock was also launched on rutin only using triangle matcher placement (60 poses, affinity‐dG score) and the induced fit refinement method (10 poses, affinity‐dG score). Final poses highlighted from the three tools were superposed in MOE using an SVL applet to obtain RMSD values with respect to both the Glide pose and the MOE pose.

## AUTHOR CONTRIBUTIONS

**Serena Francisco:** Writing – original draft; investigation; methodology; validation; visualization; data curation; formal analysis; conceptualization. **Lorenzo Lamacchia:** Methodology; investigation. **Attilio Turco:** Software; methodology. **Giuseppe Ermondi:** Resources; supervision; project administration; writing – review and editing; conceptualization; funding acquisition. **Giulia Caron:** Resources; supervision; project administration; writing – review and editing; conceptualization; funding acquisition. **Matteo Rossi Sebastiano:** Supervision; data curation; project administration; formal analysis; writing – review and editing; writing – original draft; visualization; conceptualization; validation; methodology.

## Supporting information


**Data S1.** Supporting information.
