# Supplementary material for: Restoring adapter protein complex 4 function with small molecules: an in silico approach to spastic paraplegia 50
Source: Protein Sci. 2024 Dec 26;34(1):e70006. doi: 10.1002/pro.70006 (PMC11670165; doi:10.1002/pro.70006)

**Supplementary Material**

Restoring adapter protein complex 4 function with small molecules: an *in silico* approach to SPG50

Serena Francisco^1^, Lorenzo Lamacchia^1^, Attilio Turco^1^, Giuseppe Ermondi^1^, Giulia Caron^1^, and Matteo Rossi Sebastiano^1*^

^1^ Department of Molecular Biotechnology and Health Sciences, University of Torino, Piazza Nizza 44bis, 10126 Torino, Italy.

**Correspondence**:

Matteo Rossi Sebastiano, Department of Molecular Biotechnology and Health Sciences, University of Torino, Piazza Nizza 44bis, 10126 Torino, Italy.

Email: [matteo.rossisebastiano@unito.it](mailto:matteo.rossisebastiano@unito.it)

Phone: +39 0116706370

**Table S1.** List of SPG50-associated mutations adapted from Defining the clinical, molecular and imaging spectrum of adaptor protein complex 4-associated hereditary spastic paraplegia, Ebrahimi-Fakhari et al. (2020). For clarity, we reported nonsense, frameshift and missense mutations in blue, magenta and green, respectively. The CADD (Combined Annotation Dependent Depletion) score is also reported, which provides a measure of the deleteriousness for single nucleotide variants, multi-nucleotide substitutions as well as insertion/deletions variants.

| Family/Patient | Allele 1 [cDNA] | Allele 1 [Protein] | CADD | Allele 2 [cDNA] | Allele 2 [Protein] | CADD |
| --- | --- | --- | --- | --- | --- | --- |
| 10 / 013 & 014 | c.1012C>T | p.Arg338* | 42 | c.1012C>T | p.Arg338* | 42 |
| 11 / 015 & 016 | c.952C>T | p.Arg318* | 36 | c.952C>T | p.Arg318* | 36 |
| 20 / 028, 029, 030, 031, 032 | c.1137+1G>T | p.? | 31 | c.1137+1G>T | p.? | 31 |
| 21 / 033 & 034 | c.577G>A | p.Glu193Lys | 28,7 | c.577G>A | p.Glu193Lys | 28,7 |
| 22 / 035 & 036 | c.194_195delAT | p.Tyr65Phefs*50 | 25,1 | c.194_195delAT | p.Tyr65Phefs*50 | 25,1 |
| 23 / 037 & 038 | c.1137+1G>T | p.? | 31 | c.1137+1G>T | p.? | 31 |
| 24 / 039, 040, 041 | c.521dupT | p.Leu175Profs*6 | 27 | c.955T>C | p.Cys319Arg | 25,5 |
| 25 / 042 | c.1012C>T | p.Arg338* | 42 | c.1012C>T | p.Arg338* | 42 |
| 36 / 062 & 063 | c.1137+1G>T | p.? | 31 | c.1137+1G>T | p.? | 31 |
| 41 / 069, 070, 071 | c.916C>T | p.Arg306* | 39 | c.916C>T | p.Arg306* | 39 |
| 44 / 074 | c.218dupA | p.Asn73Lysfs*43 | 24,7 | c.851A>C | p.Tyr284Ser | 30 |
| 57 / 092 | c.1026-1G>T | p.? | 33 | c.1026-1G>T | p.? | 33 |
| 58 / 093 | c.1117C>T | p.Gln373* | 41 | c.1321C>T | p.Arg441* | 39 |
| 59 / 094 | c.916C>T | p.Arg306* | 39 | c.916C>T | p.Arg306* | 39 |
| 60 / 095 & 096 | c.330C>G | p.Tyr110* | 35 | c.544-8_544-3delTTTCTC | p.? | 12,15 |
| 64 / 101 | c.32delA | p.Lys11Argfs*27 | 33 | c.955T>C | p.Cys319Arg | 25,5 |
| 68 / 106, 107, 108 | c.1137+1G>T | p.? | 31 | c.1137+1G>T | p.? | 31 |
| 70 / 110 | c.1012C>T | p.Arg338* | 42 | c.1012C>T | p.Arg338* | 42 |
| 71 / 111 & 112 | c.916C>T | p.Arg306* | 39 | c.916C>T | p.Arg306* | 39 |
| 74 / 116 & 117 | c.952C>T | p.Arg318* | 36 | c.952C>T | p.Arg318* | 36 |
| 75 / 118 & 119 | c.974+1G>T | p.? | 27,8 | c.974+1G>T | p.? | 27,8 |
| 76 / 120 & 121 | c.694delG | p.Glu232Lysfs*10 | 18,33 | c.694delG | p.Glu232Lysfs*10 | 18,33 |
| 77 / 122 & 123 | c.952C>T | p.Arg318* | 36 | c.952C>T | p.Arg318* | 36 |
| 78 / 124 & 125 | c.802C>T | p.Arg268* | 40 | c.802C>T | p.Arg268* | 40 |
| 82 / 132 | c.920G>C | p.Gly307Ala | 23,7 | c.1100G>A | p.Arg367Gln | 29,8 |
| 92 / 146 | c.1321C>T | p.Arg441* | 39 | c.956G>A | p.Cys319Tyr | 25,6 |
| 95 / 149 | c.916C>T | p.Arg306* | 39 | c.694dupG | p.Glu232Glyfs*21 | 32 |
| 96 / 150 | c.802C>T | p.Arg268* | 40 | c.802C>T | p.Arg268* | 40 |
| 98 / 152 | c.680G>A | p.Arg227His | 23,8 | c.680G>A | p.Arg227His | 23,8 |
| 99 / 153 | c.89delC | p.Glu31Serfs*7 | 22,1 | c.89delC | p.Glu31Serfs*7 | 22,1 |

**Figure S1.** AIUPred profile of order vs. disorder for AP4M1. The red line indicates the cutoff to call structured and intrinsically disordered protein regions. All residues undergoing mutation fall in structured regions, with the exception of R367.


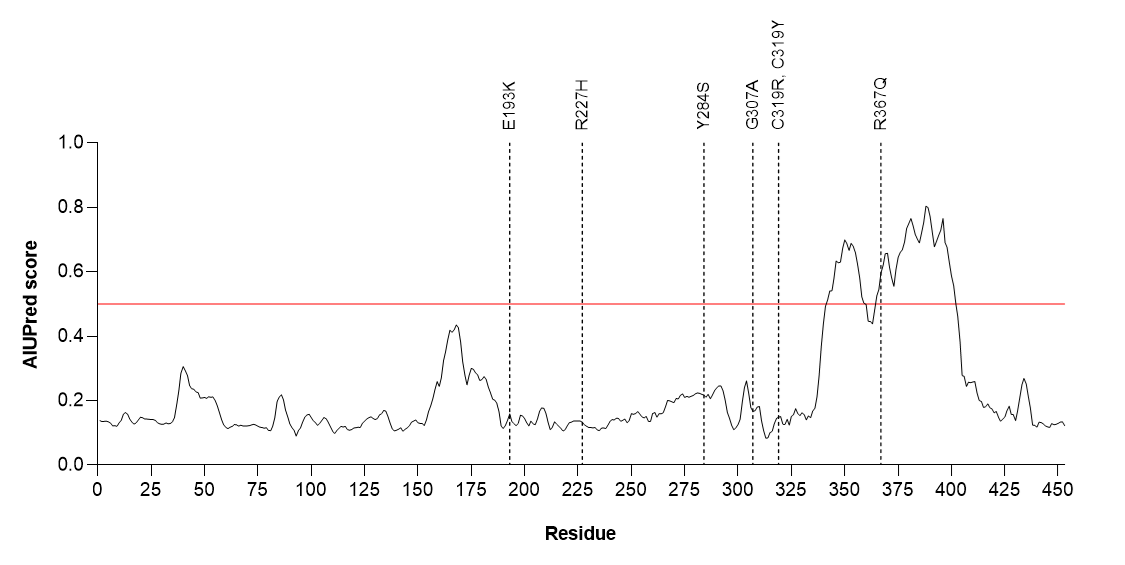


**Table S2**. Interactions established by R227, G307 and R367 in the wild type AP-4 core in closed state obtained via the MOE Contacts application.


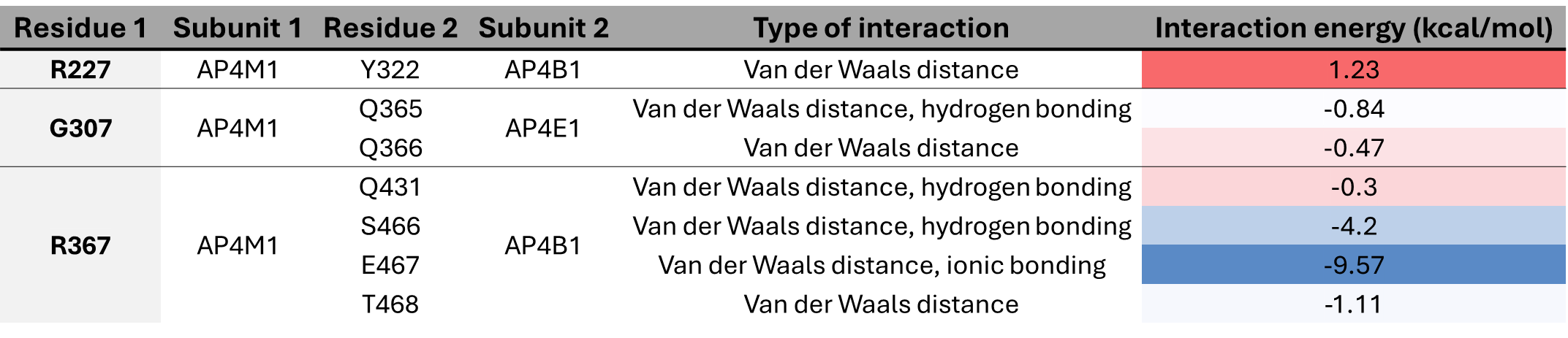


**Figure S2.** The molecular scenario of R227H, G307A and R367Q. Wild-type vs. mutant residues and their interacting partners, both in the closed and open AP-4 models, are displayed in PyMOL. Residues undergoing mutation are labelled in red in all pictures. Yellow dotted lines indicate hydrogen bonds. Magenta dotted lines represent salt bridges. AP4B1: blue; AP4M1: pink; AP4E1: green; AP4S1: grey.


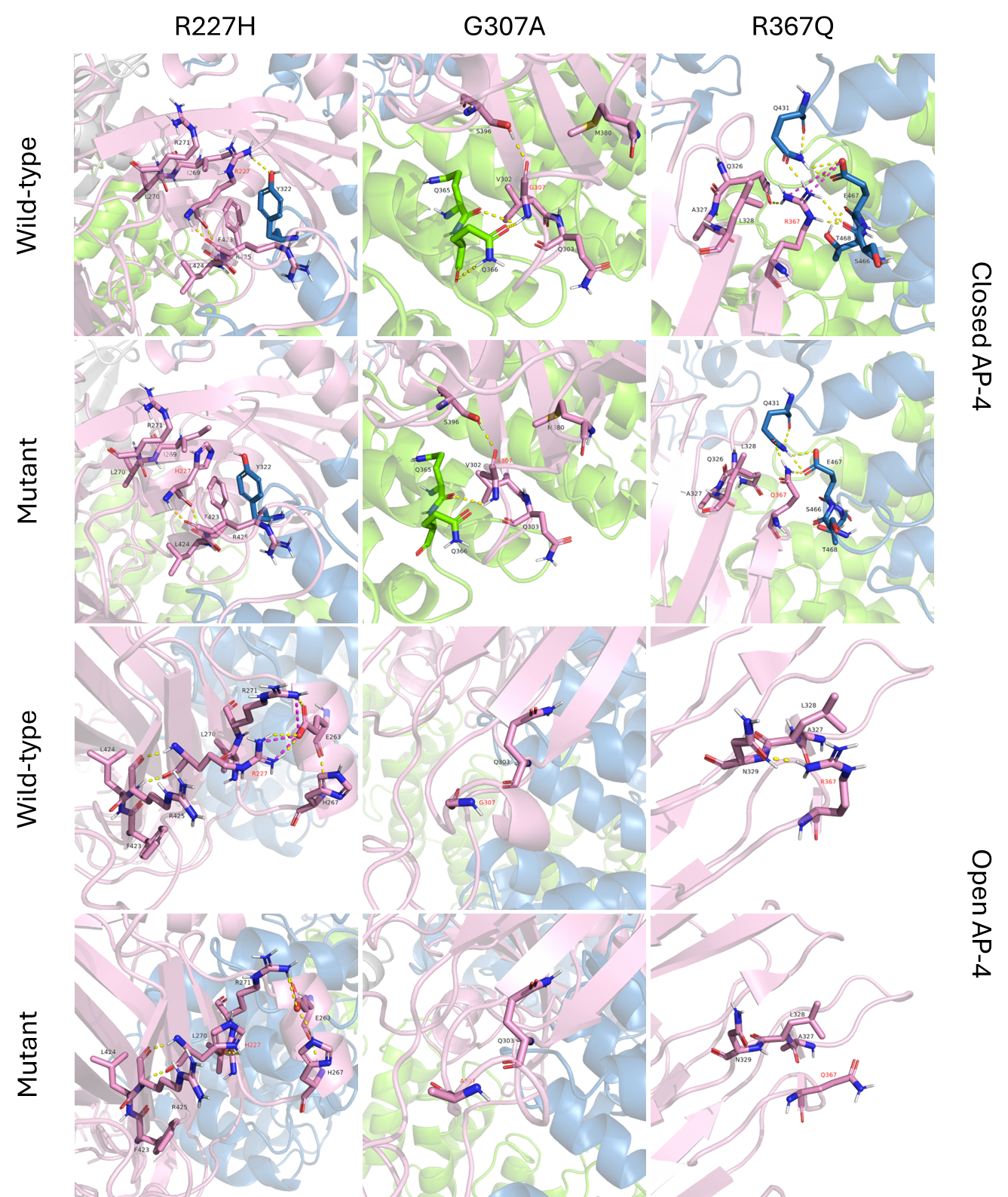


**Figure S3.** Electrostatic potential maps of the open AP-4 core computed in ChimeraX. The dashed circles highlight the co-planar electropositive patches on AP4E1 (Patch 1) and AP4M1 (Patch 2). Arginine 367 of AP4M1 is circled in green in the wild-type model. Electrostatic potential maps for the wild type (left) and R367Q mutant (right) models indicate the loss of an electropositive patch on the AP4M1 subunit upon the R367Q substitution (Patch 2).


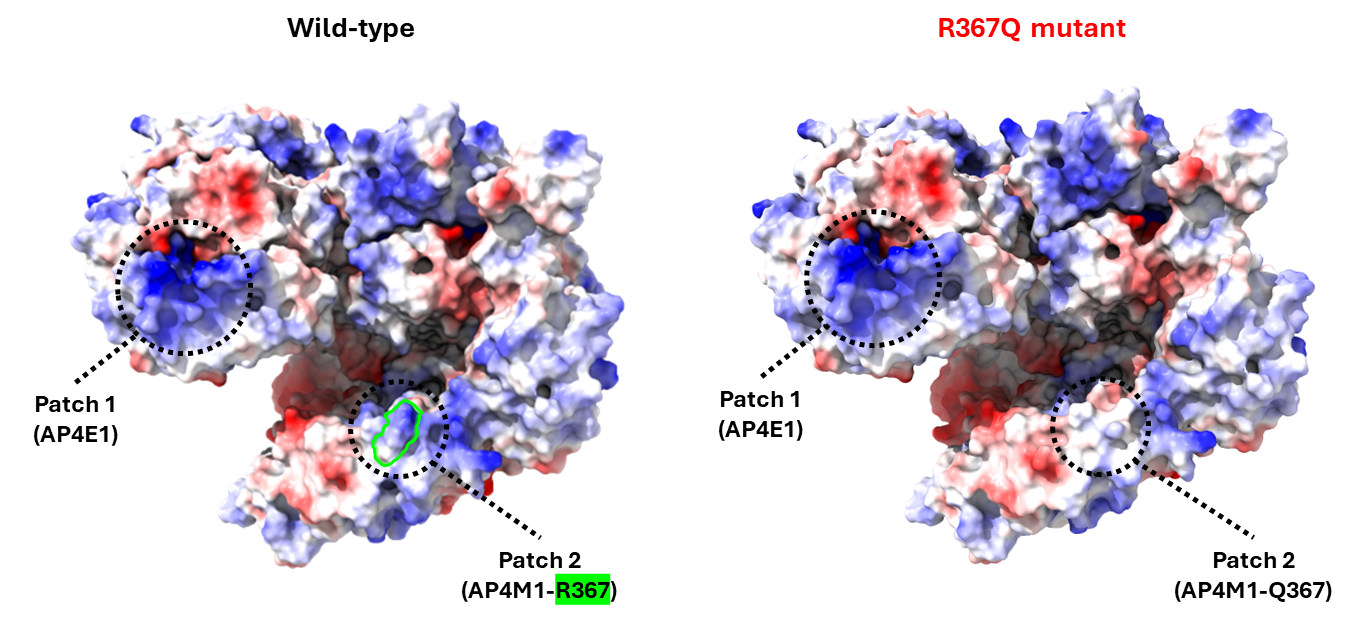


**Figure S4.** Comparison between contacts established by rutin with AP4B1 and AP4M1 according to either Glide (left figure) and MOE-Dock (right figure). Both systems were analysed from the 2D ligand interaction diagram available in Maestro. Interacting residues common to both systems encompass N429, K436, E462 (AP4B1) and E410 (AP4M1).


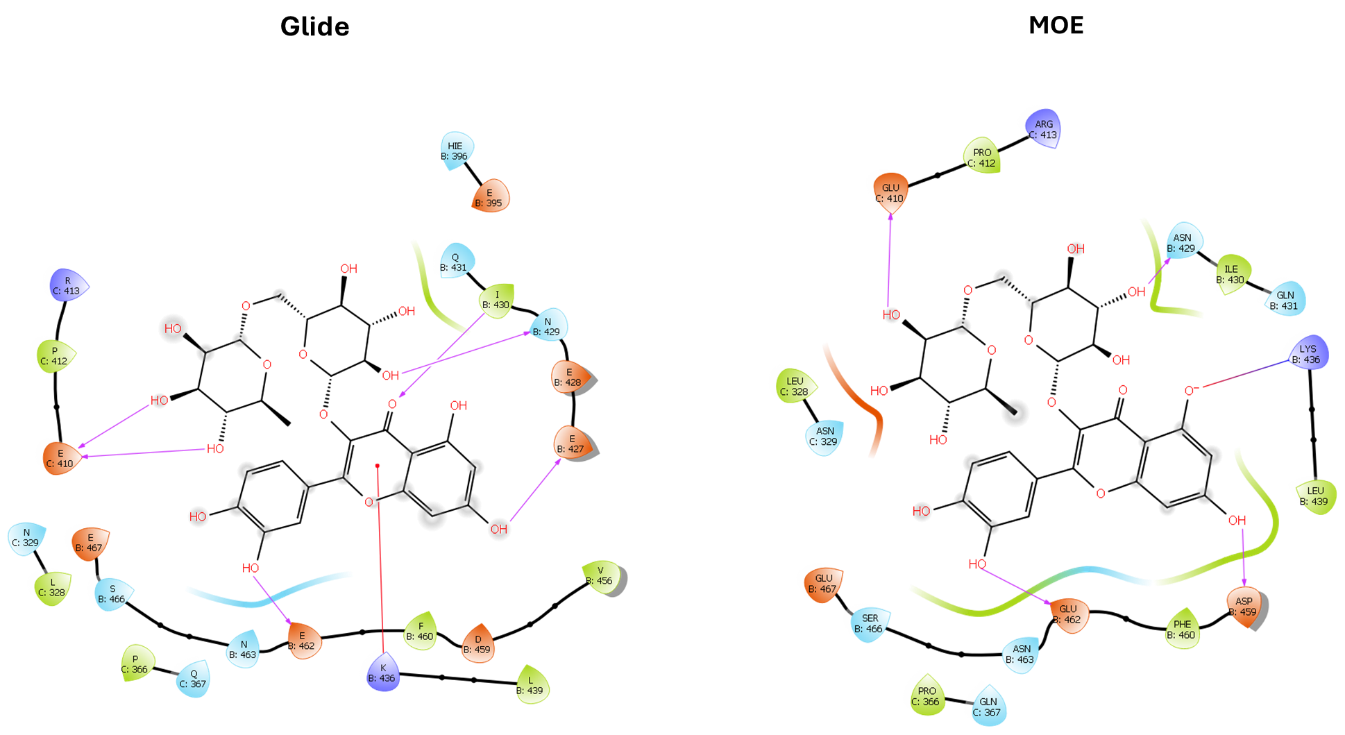

Supplement: Supplementary file 1 — Data S1. Supporting information. [file PRO-34-e70006-s001.docx]
